# Supplementary material for: Acceptability and feasibility pilot randomised controlled trial of medical skin camouflage for recovery of women prisoners with self-harm scarring (COVER): the study protocol
Source: BMJ Open. 2019 Jan 17;9(1):e021891. doi: 10.1136/bmjopen-2018-021891 (PMC6340434; doi:10.1136/bmjopen-2018-021891)
Supplement: Author's manuscript [file bmjopen-2018-021891.draft_revisions.pdf]

BMJ Open

BMJ Open is committed to open peer review. As part of this commitment we make the peer review history of every article we publish publicly available.

When an article is published we post the peer reviewers' comments and the authors' responses online. We also post the versions of the paper that were used during peer review. These are the versions that the peer review comments apply to.

The versions of the paper that follow are the versions that were submitted during the peer review process. They are not the versions of record or the final published versions. They should not be cited or distributed as the published version of this manuscript.

BMJ Open is an open access journal and the full, final, typeset and author-corrected version of record of the manuscript is available on our site with no access controls, subscription charges or pay-per-view fees (http://bmjopen.bmj.com).

If you have any questions on BMJ Open's open peer review process please email info.bmjopen@bmj.com

BMJ Open

An acceptability and feasibility pilot of medical skin camouflage for recovery of women prisoners with self-harm scarring (COVER): the study protocol.

Journal:	*BMJ Open*
Manuscript ID	bmjopen-2018-021891
Article Type:	Protocol
Date Submitted by the Author:	24-Jan-2018

Complete List of Authors:	Mitchell, Heather; University of Manchester, Centre for Women's Mental Health; Greater Manchester Mental Health NHS Foundation Trust, Psychological Research<br>Abel, Kathryn; University of Manchester, Centre for Women's Mental Health; Greater Manchester Mental Health NHS Foundation Trust, Psychological Research<br>Dunlop, Brendan; University of Manchester, Centre for Women's Mental Health; Greater Manchester Mental Health NHS Foundation Trust, Psychological Research<br>Walker, Tammi; University of Huddersfield, Department of Psychology<br>Ranote, Sandeep; North West Boroughs Healthcare NHS Foundation Trust<br>Robinson, Louise; Lancashire Care NHS Foundation Trust; University of Manchester, Centre for Women's Mental Health<br>Edgar, Fiona; University of Manchester, Centre for Women's Mental Health; University of Central Lancashire, Mental Health and Criminal Justice Group<br>Millington, Tracy; University of Manchester, Centre for Women's Mental Health<br>Meacock, Rachel; Univ Manchester,<br>Shaw, Jennifer; University of Manchester, Offender Health Research Network<br>Gutridge, Kerry; University of Manchester, Centre for Women's Mental Health

Keywords:	Medical skin camouflage, self-harm, scarring, women prisoners, recovery, feasibility trial

SCHOLARONE™ Manuscripts

An acceptability and feasibility pilot of medical skin camouflage for recovery of women prisoners with self-harm scarring (COVER): the study protocol.

Heather Mitchell, [1,2] Professor Kathryn Abel, [1,2] Brendan J Dunlop, [1,2] Dr Tammi Walker, [3] Dr Sandeep Ranote, [4] Dr Louise Robinson, [5,6] Fiona Edgar,[1, 7] Tracy Millington,[1] Rachel Meacock, [8] Professor Jenny Shaw,[9] Dr Kerry Gutridge.[1]

Heather Mitchell, BA
[1]Centre for Women's Mental Health, Division of Psychology and Mental Health, School of Health Sciences, Faculty of Biology, Medicine and Health, University of Manchester, Jean McFarlane Building, Manchester, M13 9PL and [2]Greater Manchester Mental Health NHS Foundation Trust, Bury New Road, Prestwich, M25 3BL, United Kingdom.
heather.mitchell@manchester.ac.uk

Professor Kathryn Abel, PhD, MB ChB
[1]Centre for Women's Mental Health, Division of Psychology and Mental Health, School of Health Sciences, Faculty of Biology, Medicine and Health, University of Manchester, Jean McFarlane Building, Manchester, M13 9PL and [2]Greater Manchester Mental Health NHS Foundation Trust, Bury New Road, Prestwich, M25 3BL, United Kingdom.
kathryn.abel@manchester.ac.uk

Brendan J Dunlop, BSc (corresponding author)
[1]Centre for Women's Mental Health, Division of Psychology and Mental Health, School of Health Sciences, Faculty of Biology, Medicine and Health, University of Manchester, Jean McFarlane Building, Manchester, M13 9PL and [2]Greater Manchester Mental Health NHS Foundation Trust, Bury New Road, Prestwich, M25 3BL, United Kingdom.
brendan.dunlop@manchester.ac.uk

Dr Tammi Walker, PhD
[3]Department of Psychology, School of Human & Health Sciences, University of Huddersfield, Edith Key Building, Queensgate, Huddersfield, HD1 3DH, United Kingdom.
t.walker@hud.ac.uk

Dr Sandeep Ranote, MB ChB
[4]5 Boroughs Partnership NHS Foundation Trust, Hollins Park Hospital, Hollins Lane, Winwick, Warrington, WA2 8WA, United Kingdom.

For peer review only - http://bmjopen.bmj.com/site/about/guidelines.xhtml

For peer review only

sandeep.ranote@5bp.nhs.uk

Dr Louise Robinson, MB ChB

[5]Lancashire Care NHS Foundation Trust, Preston, United Kingdom and [6]Centre for Mental Health and Risk, Division of Psychology and Mental Health, School of Health Sciences, Faculty of Biology, Medicine and Health, University of Manchester, Jean McFarlane Building, Manchester, M13 9PL, United Kingdom.

louise.robinson@manchester.ac.uk

Fiona Edgar

[1]Centre for Women's Mental Health, Division of Psychology and Mental Health, School of Health Sciences, Faculty of Biology, Medicine and Health, University of Manchester, Jean McFarlane Building, Manchester, M13 9PL, United Kingdom and [7]Mental Health and Criminal Justice Group, University of Central Lancashire, Preston, United Kingdom .

FEdgar@uclan.ac.uk

Tracy Millington

[1]Centre for Women's Mental Health, Division of Psychology and Mental Health, School of Health Sciences, Faculty of Biology, Medicine and Health, University of Manchester, Jean McFarlane Building, Manchester, M13 9PL, United Kingdom.

7milliebob@gmail.com

Rachel Meacock, MA

[8]Manchester Centre for Health Economics, Institute of Population Health, Division of Population Health, Health Services Research and Primary Care, University of Manchester, Jean McFarlane Building, Manchester, M13 9PL, United Kingdom.

rachel.meacock@manchester.ac.uk

Professor Jenny Shaw, PhD, MB ChB

[9]Offender Health Research Network, Division of Psychology and Mental Health, School of Health Sciences, Faculty of Biology, Medicine and Health, University of Manchester, Jean McFarlane Building, Manchester, M13 9PL, United Kingdom.

jennifer.j.shaw@manchester.ac.uk

Dr Kerry Gutridge, PhD

For peer review only

[1]Centre for Women's Mental Health, Division of Psychology and Mental Health, School of Health Sciences, Faculty of Biology, Medicine and Health, University of Manchester, Jean McFarlane Building, Manchester, M13 9PL, United Kingdom.

kerry.gutridge@manchester.ac.uk

Word count: 5,078

Abstract

Introduction

Self-harm in prison is a major public health concern. Less than 5% of UK prisoners are women, but they carry out more than a fifth of prison self-harm. Scars resulting from self-harm can be traumatising and stigmatising, yet there has been little focus on recovery of women prisoners with self-harm scarring. Medical skin camouflage (MSC) clinics treat individuals with disfiguring skin conditions, with evidence of improved wellbeing, self-esteem and social interactions. Only one community study has piloted the use of MSC for self-harm scarring.

Methods & Analysis

We describe an acceptability and feasibility pilot randomised controlled trial; the first to examine MSC for women prisoners who self-harm. We aim to randomise 20 women prisoners to a 6-week MSC intervention and 20 to a wait-list control (to receive the MSC after the study period). We aim to train at least 6-10 long-term prisoners with personal experience of self-harm to deliver the intervention. Pre- and post-intervention, we will pilot collection of women-centred outcomes, including quality of life, wellbeing and self-esteem. We will pilot collection of self-harm incidents during the intervention, resources used to manage/treat self-harm and follow-up of women at 12-weeks from baseline. Data on recruitment, retention and drop-out will be recorded. We aim for the acceptability of the intervention to prison staff and women prisoners to be explored in qualitative interviews and focus groups.

Ethics and dissemination

For peer review only - http://bmjopen.bmj.com/site/about/guidelines.xhtml

Informed consent will be the primary consideration; it will be made clear that participation will have no effect on life in prison or eligibility for parole. Due to the nature of the study, disclosures of serious self-harm may need to be reported to prison officials. We aim for findings to be disseminated via events at the study prison, presentations at national/international conferences, journal publications, prison governor meetings and university/NHS trust communications.

Trial Registration

The trial is registered on clinicaltrials.gov (identifier: NCT02638974).

Strengths & limitations of this study

- Several potential benefits include improved self-confidence, wellbeing and social relationships.
- Improving wellbeing may reduce likelihood of repeated self-harm, producing significant savings for the prison and NHS.
- The intervention may benefit long-term prisoners, who may find participation rewarding, and prison staff may feel better equipped to support women who self-harm.
- The nature of conducting a study within a prison raises challenges, including attrition and effective interdisciplinary co-ordination.

Key Words: Medical skin camouflage, self-harm, scarring, women prisoners, recovery, feasibility trial.

INTRODUCTION

Self-harm in Women's Prisons

Self-harm is defined as 'intentional self-poisoning or injury, irrespective of the apparent purpose of the act'[1]. The most common methods for self-harm in women's prisons are cutting and scratching followed by self-strangulation[2]. This complex behaviour is an increasing public health concern, not least because of its association with acute psychological distress and increased suicide risk[2, 3].

For peer review only

For peer review only - http://bmjopen.bmj.com/site/about/guidelines.xhtml

Self-harm is extremely prevalent and increasing in UK prisons. In the 12 months to December 2016 there were 7,657 incidents of self-harm in female prisons, an increase of 4% on the previous year[4]. This is a rate of 1,987 self-harm incidents per 1,000 prisoners. Although women make up approximately 5% of the UK prison population, they are responsible for around a fifth of all prison self-harm[4].

Research has shown that living with disfigurement from non-self-harm causes can have long-term physical and psychosocial effects, including reduced social interaction, increased social anxiety and reduced quality of life[5, 6]. Furthermore, living with scars can be challenging in a society which values physical attractiveness[7, 8]. It is likely that women prisoners with self-harm scarring experience similar psychosocial difficulties e.g. low self-esteem and interpersonal problems. These may be exacerbated by guilt and shame that women may feel because of their self-inflicted injuries[9]. There are, however, individuals who feel ambivalent about their self-harm scars, and whilst they may attempt to conceal scars in certain contexts, some feel confident and comfortable with their physical appearance[10].

Medical Skin Camouflage

Medical skin camouflage (MSC) uses British National Formulary-listed preparations to reduce the visibility of scarring or disfigurement[11], with the potential to restore self-esteem, and aid recovery[12, 13, 14]. Products include skin-matched creams and powders that are waterproof, opaque and allow adherence to textured skin. All the products are 'borderline prescription' products that are available on NHS prescription at each prescriber's discretion. A systematic review of the use of MSC in prisons yielded no available studies. Only a handful of published studies have evaluated the emotional/psychological benefits of MSC and all were in dermatological diseases or burns scarring. They report significant psychological benefit, improved social and sexual relationships and improved employability[8, 15, 16]. Despite these potential benefits, few services offer MSC for self-harm scarring[17].

For peer review only - http://bmjopen.bmj.com/site/about/guidelines.xhtml

There has been little focus on how prisoners feel about their self-harm scars and no formally evaluated interventions to help women cope with any related psychosocial difficulties. This is the first study to formally deliver and evaluate an MSC intervention in a women's prison. Potential benefits of the intervention may include 1) increased self-esteem, confidence and quality of life; 2) empowering women to take part in work and social activities they might otherwise avoid and 3) enhancing the strategies and interventions that prison staff have to work with self-harm[18]. Previous work by the research team has shown that there is a difficult relationship between prison staff and prisoners who self-harm and that staffs feel restricted in how to help women[19]. This intervention may help staff to support women with self-harm scars and promote positive staff attitudes about self-harm and its management.

This study has been developed in collaboration with staff from 5 Boroughs Partnership (5BP) NHS Foundation Trust who recently piloted an innovative camouflage service for service users with self-harm scars[20]. The 6-month pilot found that 95% of young people who used the MSC experienced improved confidence and ability to engage in activities[20]. To our knowledge, this is the first time that MSC has been evaluated in a mental health service and provided as part of a recovery package. The 5BP MSC service continues to be run in partnership with Changing Faces, a registered charity that uses volunteers to teach the MSC techniques to people in the community. This feasibility and acceptability study would provide insight into any benefits of using MSC in women's prisons and also any downsides, risks or unintended consequences.

Phase 1 and 2

The MSC intervention and protocol used in our study were informed by the Changing Faces MSC training materials[21] and modified in Phase 1 and 2 of the project. Phase 1 involved one focus group, with women prisoners with experience of self-harm (n=10) and one with prison staff (n=10). Both groups were conducted in safer custody meeting rooms and lasted between 60-90 minutes. The staff focus group explored and refined practical aspects of delivering MSC in the prison, including details of how participants would be recruited, where MSC clinics would be held and whether any MSC

For peer review only - http://bmjopen.bmj.com/site/about/guidelines.xhtml

items would be unsuitable for prison use. The focus group with women prisoners helped to select the set of women-centred outcome measures and discussed their thoughts on long-term prisoners delivering the intervention. Women said they would prefer to be trained by other prisoners, particularly other women who have self-harmed. The rationale for recruiting long-term prisoners to deliver the intervention was to improve the sustainability of the intervention since they are likely to remain in the prison for a long time and can therefore continue training women to use MSC. Women also discussed the idea of completing a weekly diary; they thought this would be a good way of recording any thoughts or incidents of self-harm and some women had used a diary previously. Phase 2 involved adapting the MSC treatment intervention based on these focus groups, and producing the training and intervention protocols. The full analysis of the focus groups will be reported in a separate paper.

Study Aims:

1) To evaluate the feasibility and acceptability of a Randomised Controlled Trial (RCT) of MSC for women prisoners with self-harm scarring.
2) To assess the feasibility and acceptability of long-term prisoners delivering the MSC intervention.
3) To test the feasibility and acceptability of collecting a set of women-centred outcome measures pre- and post-intervention, as well as a weekly self-harm diary.
4) To pilot follow-up of women at 12 weeks after baseline.
5) To test the feasibility and acceptability of collecting resource use data relating to self-harm incidents.

METHODS & ANALYSIS

Design

This study is a feasibility pilot of an RCT, incorporating a qualitative component to assess the acceptability of MSC to women prisoners and prison staff. The study is taking place in one UK closed

For peer review only - http://bmjopen.bmj.com/site/about/guidelines.xhtml

women's prison. The research is funded by the National Institute for Health Research (NIHR) Research for Patient Benefit Programme (PB-PG-1013-32075). It was approved by West of Scotland Research Ethics Committee (16/WS/0155). The current protocol version is Version 6 (17/05/2017).

Sample size

Over 6 months (January 2017 – May 2017), we aim to recruit at least 6-10 long-term women prisoners to be trained in MSC. These women will then deliver the intervention to trial participants. The long-term prisoners will not be participants in the RCT, but will instead form an integral part of the research team delivering the intervention to the RCT participants.

Over eight months (January 2017 – September 2017) we aim to recruit and consent 40 women prisoners to be randomised to receive either MSC or 'treatment as usual' (TAU) in a wait-list control (to receive the MSC after the study period) design. Based on previous research[22] and recent figures from the study prison, we estimate that there will be around 5-6 eligible women per month.

Participants and recruitment procedures

Recruitment procedures and advertisement strategies have been informed by the Phase 1 focus groups. The research team will advertise the research at Safer Custody meetings attended by women prisoners. Leaflets and posters will be distributed to our local collaborators in the prison for display in different locations around the prison. Women will inform the local collaborators if they are interested in participating. We have one member of Safer Custody staff collaborating with the researchers and a woman prisoner from the Safer Custody team organising the research appointments. Prison staff will assess all volunteers to determine whether they would pose a risk to the researchers or other participants. Staff will also check the woman's sentence length to ensure she has enough time remaining on her sentence to take part in the study. In addition, healthcare staff will review the list of women to assess whether there are any health reasons why they might not be safe to participate. This has been usual practice across our decade of prisoner participation in research.

For peer review only - http://bmjopen.bmj.com/site/about/guidelines.xhtml

Vetted/screened women will be provided with an information sheet and offered the opportunity for the research team to visit, read through the sheet and answer any questions. Consent for the research will be agreed at least 24 hours after the information sheet has been read.

Inclusion/exclusion criteria

Phase 3:

We aim to recruit 6-10 long-term prisoners with at least 10 years or more left on their sentence and who have experience of self-harm.

Discussions with prison staff suggested that the most suitable long-term women would be those who already hold a position of responsibility in the prison e.g. a peer supporter or trained Samaritan listener.

Phase 4:

We aim to recruit 40 women prisoners screened for date of release, with sufficient time left on their sentence to complete the intervention period. The women will have self-harm scarring anywhere on their body that they are happy to show to others, with at least some closed wounds (to allow the MSC to be applied).

All participants (phase 3 and 4) will be aged 18 or older and able to give written, informed consent. Capacity to consent will be assessed by the experienced researchers (HM and KG) in collaboration with Safer Custody and Mental Health Care contacts in the prison. Participants will be excluded from the study if they are unable to provide written informed consent, or if they pose a risk to researchers (as assessed by the prison).

Randomisation

In Phase 4 internet randomisation (using an internet-based programme to randomise participants; www.sealedenvelope.com) will be carried out by the non-blind members of the research team, KG or HM, to allocate eligible women to MSC or waitlist control. Women in the waitlist group would

For peer review only - http://bmjopen.bmj.com/site/about/guidelines.xhtml

receive one skin-matched prescription of MSC at the end of the research. Waitlist control has been chosen as the comparator to give all participants an opportunity to use the MSC. Participants randomised to the waitlist control would be aware of their allocation (figure 1).

The Intervention – Medical Skin Camouflage for Self-harm scarring (adapted for delivery in a women's prison)

Development

Outcomes of the Phase 1 focus groups informed the development of the MSC intervention materials. In addition, two service user researchers (FE and TM) provided guidance to the research team, focusing on whether the intervention materials were suitable in terms of readability and sensitivity.

The intervention package consists of the training manual and four additional documents. The main training manual has been adapted from training manuals used by Changing Faces[21]. The adapted materials have been reviewed by a representative from the charity, to ensure that all key learning and safety points are covered.

Manual Content

The 34-page training manual has 13 sections that are listed and briefly described in Table 1 below.

Table 1: Sections of the training manual

Section Number	Section Name	Overview	Key Learning Point
1	Self-harm	This section aims to help women understand the different forms that self-harm can take and different reasons why women self-harm.	Different people have very different reasons for self-harming and it is therefore important to not make assumptions.
2	Working with women who self-harm in the COVER project	This section covers how to manage confidentiality and how to work with women who self-harm e.g. being respectful, don't judge the participant, the limits of confidentiality.	To manage and understand the limits of confidentiality, e.g. if she discloses something that puts her or someone else at risk, and what to do if a woman becomes upset.

For peer review only - http://bmjopen.bmj.com/site/about/guidelines.xhtml

3	Hygiene	This section covers how to run a hygienic skin camouflage clinic and how to keep the kit clean.	Hygiene rules to follow during an appointment.
4	Communication	This section covers communication rules, including how to manage participant expectations e.g. setting realistic expectations for what MSC can achieve.	Understanding the importance of helping the client to express their wishes and working with them to achieve the best results.
5	The Skin & Skin types	An overview of preparing the skin for application of MSC and how to ensure safe usage e.g. by checking for allergies.	How to prepare the skin and when it is not safe to use the products.
6	Overview of the kit	This section describes the items in the MSC kits and how to lay them out in a logical order.	Laying the kit out in a logical order will help the practitioner to quickly identify the products.
7	Colour Matching	This section covers colour matching. This will involve some practical activities on identifying colour tones and colour matching.	To be able to identify tones in the creams and perform a colour match.
8	Brush Technique	An overview of the brush technique and when/how to use it.	To understand when and how to use brushes.
9	Finger Technique	An overview of the finger technique and when/how to use it.	To understand when and how to use the finger technique.
10	Sponge Technique	An overview of the sponge technique and when/how to use it.	To understand when and how to use sponges.
11	Spreading Technique	An overview of the spreading technique and when/how to use it.	To understand when and how to use the spreading technique.
12	Working with powder	An overview of how to use powder to set the MSC creams.	To understand the purpose of powder, and how to apply it.
13	Completing the record card	This section covers how to complete the participant record card, including what to do with the record card after the appointment.	What to include on the record card.

Accompanying Documents

1) A single sheet of key learning points for long-term prisoners covering safety issues such as how to protect trial participants e.g. breaking confidentiality if a woman discloses something which suggests she or someone else is at risk of harm.
2) A monitoring sheet for long-term prisoners to be used in weekly meetings with the research team. The form will help to identify whether any further training or support is required.

For peer review only - http://bmjopen.bmj.com/site/about/guidelines.xhtml

3) An appointment checklist for long-term prisoners breaking down the 14 core steps in a MSC appointment, from laying out the kit, to completing a prescription record card.
4) A DOs and DON'Ts sheet for trial participants: this covers reminder points, including those related to safety and hygiene (e.g. always keep lids on the products) and some rules relating to continued participation in the trial (e.g. don't trade or share the products as only one prescription will be provided, added at the request of prison staff).

Delivery of the Intervention

Three stages of delivery: 1) training sessions for long-term prisoners, 2) skin camouflage clinics run by long-term prisoners for trial participants, 3) prescription of MSC products by prison healthcare.

1) Members of the research team aim to deliver a half-day training session to 6-10 long-term women prisoners. During this session, the research team will work through the training manual, answering any questions and giving practical demonstrations of colour matching, application techniques and powdering. Participants will participate in practical activities to ensure that they have understood the training and are competent in MSC. There is scope for the training time to be extended if the women require more practice.

2) The aim is that regular skin camouflage appointments will be run by the trained long-term prisoners. The appointments will be held during the core prison day and will not interfere with the women's income. All participants will be seen individually for one hour; the intervention group will be seen as soon as possible after they have been randomised and the waitlist control group will be seen after they have completed their 12-week follow-up. During this appointment, the long-term prisoner will provide the woman with information about the MSC creams and powders; including allergy checks to ensure the woman can safely use the products. The long-term prisoner will then perform a colour-match for the participant and demonstrate the application techniques. The participant will then practice applying the camouflage creams themselves until they are happy with the results. The long-term prisoner will then complete a record form to be given to healthcare.

For peer review only - http://bmjopen.bmj.com/site/about/guidelines.xhtml

3) The aim is for a nurse prescriber from healthcare to meet with all participants (the intervention group at the start and the waitlist control group at the end of the research) and write a prescription for 1x camouflage cream and 1x camouflage powder. Women will be informed that they will only be given one prescription for the duration of the study. The amount of camouflage cream required will depend on the extent of the participant's scarring, but based on the 5BP pilot[20] we anticipate that one prescription will be enough for a 3-month period.

Continued provision of the MSC products post-trial is not envisaged at this stage within the study prison. However, all participants will be given a letter that they have the option to give to their General Practitioner (GP) in the community that will detail their MSC prescription, and will recommend that the product is prescribed to them.

Assessing feasibility and acceptability

We will assess the feasibility of recruiting and randomising women to MSC vs wait list and of long-term prisoners delivering MSC appointments. We will examine use of the MSC, attrition (number of drop-outs at each time point), and retention (the proportion of participants who complete the intervention period). The feasibility of delivery in a prison setting (i.e. location, duration of training, peer-delivery) and the acceptability of the intervention to women and staff will be assessed using qualitative interviews and focus groups. The feasibility of undertaking a full-scale RCT of MSC for women in prison will be assessed by studying recruitment (the proportion of eligible participants consenting to join the study) and completeness of outcome measures at baseline, post-intervention (approximately nine weeks from baseline to include the time taken to receive the MSC and 6 weeks of MSC use) and at follow-up (12 weeks from baseline). We have included a 12-week follow up to assess retention and attrition over a longer period of time. Data will be collected on reasons for ineligibility, non-consent and dropout, including when the participant dropped out/withdrew from the study.

Outcome measures for future RCT design

For peer review only - http://bmjopen.bmj.com/site/about/guidelines.xhtml

For peer review only

The aim is that all participants in both groups (MSC and waitlist control) will be asked to complete a set of quantitative outcome measures at baseline (zero weeks), post-intervention (approximately nine weeks later) and at follow-up (approximately 12 weeks from baseline). This will help us to assess the feasibility and acceptability of these measures for a future clinical and cost-effectiveness RCT. Outcome measures will be administered by the project manager (PM), trained research assistant (RA) or research nurses from the NIHR clinical research network. The PM and the RA will be unblinded to the randomisation outcome and will therefore only administer baseline measures; administration of measures at any other time point by these individuals may bias results. The research nurses will be blinded and will complete the post-intervention and 12-week follow-up assessments. All research assessments (which we anticipate will last approximately 1 hour) will take place in a private room in Safer Custody. The PM, RA or research nurse will complete a case report form for each participant; recording any additional notes on each participant e.g. reasons for questionnaire non-completion. Given the sensitive nature of some of the selected outcome measures, we have consulted with women, Safer Custody staff and healthcare/mental health staff to develop procedures to protect and support participants. If, at any point during a research assessment the woman becomes agitated or distressed, we will ask them if they would like to take a break or if they want to resume the assessment on another day. If the researcher has any concerns for the woman, they will alert the local collaborator who will ensure it is dealt with accordingly using existing prison support systems. The participant information sheet outlines that the researcher is obligated to inform the prison if there is a risk to the participant's health, safety or wellbeing. For this study this will include reporting high suicidal ideation and high risk of serious self-harm.

We aim to administer a selection of outcome measures (see Table 2) to all participants at baseline, 9-weeks and 12-weeks after baseline. Two of these measures, the Dermatology Quality of Life Index (DQLi)[23] and Rosenberg Self-esteem Scale (RSES)[24], were added following focus group discussions on the psychological and interpersonal impact of scars. At baseline, we also aim to use a bespoke demographic and personal history questionnaire to collect relevant personal information including age, ethnicity, whether they are on remand or sentenced, past experience of contact

For peer review only - http://bmjopen.bmj.com/site/about/guidelines.xhtml

psychiatric services, drug dependence and experiences of domestic violence, sexual abuse and parental neglect. We aim to collect this information to check whether the two randomised groups have similar backgrounds. With women's permission, our local collaborator or a research nurse will access information on key forensic and clinical characteristics from CNomis, SystemOne (the prison electronic medical records) and from Assessment, Care in Custody and Teamwork (ACCT) documentation; these systems will be accessed by prison staff unless the researchers are granted access permission. Forensic characteristics will include types of offence (violent or non-violent), sentence length and stage of sentence and clinical characteristics will include psychiatric diagnosis and history. We aim to administer the Deliberate Self-Harm Inventory (DSHI)[25] at baseline and follow-ups: a 17-item questionnaire that assesses the history and frequency of self-harming behaviours. We also aim to administer the Zanarini Rating Scale for Borderline Personality disorder (ZAN-BPD) at all time points as a measure of borderline psychopathology[26].

Table 2: Participant Assessment Schedule

Assessment Tool	Brief Description	Time Point			
		Duration (min)	Baseline	Post-intervention	Follow-up (12 weeks)
Personal History Questionnaire	Socio-demographic/life history	5	X		
DSHI	Methods/history of self-harm	10	X	X	X
WEMWBS	Mental wellbeing	5	X	X	X
BSSI	Suicidal ideation	10	X	X	X
BDI-II	Depression	10	X	X	X
BHS	Hopelessness	5	X	X	X
DQLi	Self-harm scarring quality of life	5	X	X	X
RSES	Self-esteem	5	X	X	X
ZAN-BPD	Borderline personality disorder	5	X	X	X
EQ-5D-5L	Generic health	5	X	X	X
SF12	Generic health/quality of life	5	X	X	X
Qualitative Interview	Acceptability and feasibility	30			X
Total time burden			70	65	95
Self-harm Diary	Self-harm thoughts and incidents	Weekly from baseline to 12 weeks			

We aim to examine whether the Warwick-Edinburgh Mental Well-Being Scale (WEMWBS)[27] is a suitable primary outcome for a full-scale RCT. The WEMWBS is a 14-item scale of mental wellbeing

For peer review only - http://bmjopen.bmj.com/site/about/guidelines.xhtml

covering subjective wellbeing and psychological functioning, in which all items are worded positively and address aspects of positive mental health. The WEMWBS has high internal consistency (α = .91) and test-retest reliability (0.83)[27]. This measure would be used to calculate study power in a full-scale subsequent trial.

Becks Scale for Suicidal Ideation (BSSI)[28]: a 19-item instrument measuring intensity, duration and specificity of thoughts about committing suicide. The BSSI has high internal consistency (0.89) and high inter-rater reliability (0.83)[28]. The BSSI has been successfully used in a pilot trial of Psychodynamic Interpersonal Therapy for women prisoners who self-harm[22].

Becks Depression Inventory (BDI-II)[29]: a 21-item scale measuring symptoms of depression. The BDI-II has high internal consistency and a test-retest reliability ranging from 0.73 to 0.96[30].

Beck Hopelessness Scale (BHS)[31]: a 20-item self-report inventory designed to measure three major aspects of hopelessness: feelings about future, loss of motivation and expectations. The BHS has high concurrent validity (0.86) and high reliability (α = 0.91)[31].

Prison-adapted Dermatology Quality of Life Index (DQLi)[23]: a 7-item questionnaire adapted from a validated 10 item scale that has been used in over 40 different skin conditions in over 80 countries. Test-retest reliability has been found to be high (0.99)[23].

Rosenberg Self-Esteem Scale (RSES)[24]: a 10-item Likert scale with items answered on a four-point scale – from strongly agree to strongly disagree. The scale measures self-esteem and has been used in prison research[32]. Internal consistency ranges from 0.77 to 0.88 and test-retest reliability ranges from 0.82 to 0.85[24].

For peer review only - http://bmjopen.bmj.com/site/about/guidelines.xhtml

EQ-5D-5L[33]: a generic preference-based measure covering five domains of health-related quality of life (mobility, self-care, usual activities, pain/discomfort, anxiety/depression). Test-retest reliability is high and ranges from 0.78 to 0.87, with convergent validity at 0.64[34].

SF-12 is a shortened version of the SF-36[35], consisting of twelve questions covering eight dimensions of health: physical functioning, role limitations - physical, bodily pain, general health, vitality, social functioning, role limitations - emotional, and mental health. Test-retest reliability ranges from 0.76 to 0.89 and relative validity ranges from 0.43 to 0.93 [34].

To reduce attrition, we aim to seek consent at baseline for women who have been transferred or have left prison during the study period to be followed up in person at other prisons or in a public place in the community, following a lone worker policy.

In addition to the outcome measures listed above, we also aim to ask trial participants to complete a weekly diary every week from their baseline assessment. Prison staff and women prisoners in the Phase 1 focus groups proposed the use of a weekly diary; some of the women had completed a diary of self-harm thoughts and events in the past and found it helpful. The research team will collect the diary each week. The diary will ask questions about any thoughts or acts of self-harm that have occurred during the week and any life events that have impacted on their self-harm during the week. Women will also have a free-text space to add additional comments.

We also aim to pilot the collection of resource use data. This will be collected using the Secure Facilities Service Use Schedule (SFSUS)[36] and a bespoke resource use questionnaire. Resource use data is likely to be extracted by the local collaborator from systems such as CNomis and Officers logs. Prison staff will redact any confidential information. We also aim to use these systems, together with SystemOne, to extract data on self-harm incidents that occurred during the intervention. If we successfully extract the data we will then triangulate prison records of self-harm incidents with

For peer review only - http://bmjopen.bmj.com/site/about/guidelines.xhtml

women's self-reported incidents. We will record the time taken by prison staff and healthcare staff to extract this information.

To inform a future cost analysis, we also aim to record the time spent by Changing Faces training the researchers in medical skin camouflage, time spent by the research team training long-term prisoners to become skin camouflage practitioners, time spent by long-term prisoners delivering the intervention, and quantities of MSC products prescribed.

Qualitative Data

We aim to conduct interviews with all women in the MSC group (n=20) at the end of the study, to assess the acceptability of the intervention to service users. The interviews with women will explore their views on applying MSC, how long it stays on for, how useful they found it and any positive or negative effects on their everyday life, mood, self-esteem and self-confidence. The topic guides have been developed in consultation with two service user researchers and informed by outcomes of the Phase 1 focus group.

We also aim to interview the long-term prisoners to assess their experiences of being an MSC practitioner, in terms of the acceptability of the training, mentoring/support from the research team and any benefits or difficulties working with participants.

In addition, we aim to conduct a focus group with prison staff from different disciplines (including Safer Custody staff, prison officers and healthcare staff) that have been in contact with women involved in the trial. The focus group would explore acceptability of the intervention from a staff perspective, including what they thought about prisoner-delivery of the MSC intervention and whether the intervention has had a positive, or negative, impact on their job or their relationships with women prisoners. All interviews and the focus group will use semi-structured topic guides with open-ended questions that should enable us to explore in-depth the aspects of the intervention that worked well, the aspects that did not work well, and things that could be improved. With permission from

For peer review only - http://bmjopen.bmj.com/site/about/guidelines.xhtml

participants, interviews will be audio-recorded. All recordings will then be transcribed verbatim and analysed using thematic analysis[37].

We aim to assess fidelity to the MSC intervention by a) observing the long-term prisoners at the end of training covering one of our service user researcher's scars; b) audio recording 10% of the training sessions which will be rated for fidelity to the training manual by an independent researcher.

Data Analysis

Quantitative analyses

We shall compare means before and after treatment using descriptive statistics, including standard deviations and confidence intervals for outcome variables to inform sample size estimates for a future RCT. We will also present descriptive statistics on recruitment and retention of participants in both groups, including reasons for dropout at different stages.

We shall assess the feasibility and relevance of both the EQ-5D-5L and SF-12 for the prison population through correlation between changes from baseline to follow-up of these and other piloted measures (WEMWS; BSSI; BDI; BHS; RSES; ZAN-BPD); and examination of completion rates. Descriptive analysis of Health Related Quality of Life data will also inform the suitability of the measures for future clinical and economic evaluations of the intervention.

Resource use collection will also be assessed through time taken to complete questionnaires, completion rates and ability to obtain included resource-use categories to inform suitability of resource use categories in a future economic evaluation. Descriptive analysis of resource use data will also inform future trial design.

Qualitative data

Qualitative data will be analysed using thematic analysis[37]; analysis which will be conducted by the RA and PM and checked for accuracy by an independent researcher. Preliminary codes and categories

For peer review only - http://bmjopen.bmj.com/site/about/guidelines.xhtml

are assigned to the text[38] and emergent themes subject to constant comparison and examined for goodness-of-fit until a final set of key themes identified[39]. Adopting an inductive, iterative approach, data analysis will commence with the first interview.

Data Entry & Storage

Written consent forms and completed questionnaires will be removed straight to the University of Manchester. Participants will be given a unique participant number that will be used on questionnaires and the electronic database. A password-protected document will link participant names and numbers. Any identifying personal data (e.g. consent forms) will be stored separately from other research data. In the University of Manchester this will mean storage in the locked limited access corridor. Electronic databases will be stored on an encrypted space on University of Manchester computers. The RA would enter all data and the PM will carry out 10% checks for accuracy.

ETHICS & DISSEMINATION

Adverse events

All participants will be women who have a history of self-harm. Therefore, self-harm incidents are an expected event and not necessarily a serious adverse event. All adverse events, including incidents of self-harm, will be recorded and reported to the project manager. In consultation with prison staff and the prisoner, the research team will assess the seriousness of the adverse event and whether it is related to project participation; events that are judged as serious and unrelated will be reported to the sponsor only. Events judged as serious and related to project participation will be reported to the research sponsor, host NHS trust and West of Scotland Research Ethics Committee (REC).

Dissemination

We aim for our findings to be disseminated to prisoners, prison staff and to the wider stakeholder (academic and clinical) community via showcase events at the study prison, presentations at national and international conferences, journal publications, safer custody and prison governor meetings and university/NHS trust communications.

For peer review only - http://bmjopen.bmj.com/site/about/guidelines.xhtml

Discussion

Despite the large number of women in prison whom self-harm (or who have self-harmed in the past and are living with scarring), there are little/no evidence-based interventions which aim to improve self-esteem, confidence and wellbeing. This low-cost intervention has the potential. to improve women's mood and how they feel about themselves.

Our Phase 1 focus groups suggested that many women prisoners who repeat self-harm struggle on a regular basis with negative feelings about their scars e.g. they have to cover them in front of others/ family for fear of being judged adversely or upsetting them; they are a constant reminder of bad times or they lack confidence in their bodies because of scars. A prisoner-delivered MSC intervention could reduce such distress women prisoners experience and help them re-integrate into the community without the additional burden of being judged because of their scars.

This intervention was implemented successfully in a community mental health service. We, therefore, anticipate that, with the support of prison staff and long-term prisoners, COVER will provide a beneficial resource to improve wellbeing in an often-neglected population.

Engaging long-term prisoners in the delivery of MSC clinics should increase the sustainability of the intervention if it were to be commissioned in future and provide meaningful work for women prisoners, offering a valuable opportunity to improve relationships between prisoners and contribute towards a therapeutic community with the prison. Peer support schemes, such as the Samaritan's Listener scheme which runs across many UK prisons are increasingly popular, enabling prisoners to develop a range of transferable skills and reducing the burden of distress and self-harm management for prison staff. If successfully implemented, COVER will run alongside these peer support services and provide additional help for women who self-harm.

Authors' contributions

KA and TW conceived and designed the study and applied for funding. KG drafted the original protocol. KA, KG & HM led the development of the prison-modified MSC intervention; are

For peer review only - http://bmjopen.bmj.com/site/about/guidelines.xhtml

responsible for drafting and revising the protocol manuscript; have given final approval for the version to be published and are accountable for all aspects of the work. HM and BD led the write-up of the protocol manuscript under the supervision of KG. KG is the research project manager and HM/BD are the research assistants on the study. KA, KG, HM, JP, TW, SR, LR, JS & RM co-led the development of COVER and participated in the design of the study. KA and JS provide the senior academic oversight on all aspects of the feasibility study. KG, HM and BD lead the Patient and Public Involvement (PPI). FE and TM have provided expert by experience input throughout the project. All authors read and approved the final protocol manuscript.

Acknowledgements

We would like to thank the service users, prison staff and healthcare professionals who participated in the focus groups during phase one of the study. We would like to thank the COVER project steering group for their ongoing input and support. We would also like to thank the prison governor and our local collaborators in the prison for their support.

Funding

This project was funded by the National Institute for Health Research (NIHR)

Research for Patient Benefit Programme (reference PB-PG-1013-32075). This article presents independent research funded by the NIHR. The views and opinions expressed therein are those of the authors and do not necessarily reflect those of the RfPB programme, NIHR, NHS or the Department of Health. The study sponsor is The University of Manchester and the host NHS Trust is Greater Manchester Mental Health NHS Foundation Trust.

Competing Interests

The authors declare that they have no competing interests.

Consent for Publication

Not applicable.

For peer review only - http://bmjopen.bmj.com/site/about/guidelines.xhtml

Ethical Approval and Consent to Participate

Ethical approval has been granted by the West of Scotland Research Ethics Committee 3, REC reference: 16/WS/0155. Recruitment is currently ongoing; to date, 30 participants have entered the Phase 4 trial.

Availability of Data and Materials

Not applicable

Author Details

[1]Centre for Women's Mental Health, Division of Psychology and Mental Health, School of Health Sciences, Faculty of Biology, Medicine and Health, University of Manchester, Jean McFarlane Building, Manchester, M13 9PL. [2]Division of Psychology and Mental Health, School of Health Sciences, Faculty of Biology, Medicine and Health, University of Manchester, Jean McFarlane Building, Manchester, M13 9PL. [3]Department of Psychology, School of Human & Health Sciences, University of Huddersfield, Edith Key Building, Queensgate, Huddersfield, HD1 3DH. [4]5 Boroughs Partnership NHS Foundation Trust. [5]Lancashire Care NHS Foundation Trust, Preston, United Kingdom. [6]Centre for Mental Health and Risk, Division of Psychology and Mental Health, School of Health Sciences, Faculty of Biology, Medicine and Health, University of Manchester, Jean McFarlane Building, Manchester, M13 9PL. [7]Mental Health and Criminal Justice Group, University of Central Lancashire, Preston, UK. [8]Manchester Centre for Health Economics, Institute of Population Health, Division of Population Health, Health Services Research and Primary Care, University of Manchester, Jean McFarlane Building, Manchester, M13 9PL. [9]Offender Health Research Network, Division of Psychology and Mental Health, School of Health Sciences, Faculty of Biology, Medicine and Health, University of Manchester, Jean McFarlane Building, Manchester, M13 9PL.

Figure 1. Consort diagram.

References

1 National Collaborating Centre for Mental Health. Self-Harm: The Short-Term Physical and Psychological Management and Secondary Prevention of Self-Harm in Primary and Secondary Care. London, England: National Institute for Clinical Excellence, 2004.

For peer review only - http://bmjopen.bmj.com/site/about/guidelines.xhtml

2 Hawton K, Arensman E, Townsend E, et al. Deliberate Self Harm: Systematic Review of Efficacy of Psychosocial and Pharmacological Treatments in Preventing Repetition. *Brit Med J* 1998;317(7156):441-7 doi:10.1136/bmj.317.7156.441 [published 15 August 1998].

3 Marzano L, Fazel S, Rivlin A, et al. Psychiatric disorders in women prisoners who have engaged in near-lethal self-harm: case–control study. *Br J Psychiatry* 2010;197:219-26 doi: 10.1192/bjp.bp.109.075424.

4 Ministry of Justice. Safer in custody statistics England and Wales: Deaths in custody to March 2016, Assaults and self-harm in custody to December 2015. London: Ministry of Justice, 2016.

5 Van Loey N, Van Son M. Psychopathology and psychological problems in patients with burn scars: epidemiology and management. *Am J Clin Dermatol* 2003;4:245-72 doi:10.2165/00128071-200304040-00004.

6 Brown B, McKenna S, Siddhi K, et al. The hidden cost of skin scars: quality of life after skin scarring. *J Plast Reconstr Aesthet Surg Open* 2008;61:1049–58 doi:10.1016/j.bjps.2008.03.020 [published 9 July 2008].

7 Rumsey N, Clarke A, Musa M. Altered Body Image: The Psychosocial Needs of Patients. *Br J Community Nurs* 2002;7(11):563-6 doi:10.12968/bjcn.2002.7.11.10886 [published November 2002].

8 Levy L, Emer J. Emotional Benefit of Cosmetic Camouflage in the Treatment of Facial Skin Conditions: Personal Experience and Review. *Clin Cosmet Investig Dermatol* 2012;5:173-82 doi: 10.2147/CCID.S33860 [published 1 November 2012].

9 Leibenluft E, Gardner D, Cowdry R. The inner experience of the borderline self-mutilator. *J Pers Disord* 1987;1:317-24 doi:10.1521/pedi.1987.1.4.317.

10 Chandler A. Narrating the self-injured body. *Med Humanit* 2014;40:111-16 doi:10.1136/medhum-2013-010488.

11 McMichael L. Skin Camouflage. *Brit Med J* 2012;344:d7921 doi:10.1136/bmj.d7921 [published 5 January 2012].

For peer review only - http://bmjopen.bmj.com/site/about/guidelines.xhtml

12 Holme S, Beattie P, Fleming C. Cosmetic Camouflage Advice Improves Quality of Life. *Br J Dermatol* 2002;147(5):946-9 doi:10.1046/j.1365-2133.2002.04900.x [published 1 November 2002].

13 Green L. The Effect of Skin Conditions on Patients' Quality of Life. *Nurs Stand* 2010;25(9):48-56 doi:10.7748/ns2010.11.25.9.48.c8078 [published 03 November 2010].

14 Kent G. Testing a Model of Disfigurement: Effects of a Skin Camouflage Service on Well-Being and Appearance Anxiety. *Psychol Health* 2002;17(3):377-86 doi:10.1080/08870440290029601 [published online 27 October 2010].

15 Tanioka M, Yamamoto Y, Kato M, et al. Camouflage for Patients with Vitiligo Vulgaris Improved their Quality of Life. *J Cosmet Dermatol* 2010;9(1):72-5 doi:10.1111/j.1473-2165.2010.00479.x [published 22 February 2010].

16 Hayashi N, Imori M, Yanagisawa M, et al. Make-up Improves the Quality of Life of Acne Patients without Aggravating Acne Eruptions during Treatments. *Eur J Dermatol* 2005;15(4):284-7.

17 McConochie L, Pearson E. Development of a Nurse-Led Skin Camouflage Clinic. *Nurs Stand* 2006;20(24):74, 6, 8 passim doi:10.7748/ns.20.24.74.s59 [published February 2006].

18 Walker T, Shaw J, Ranote S, et al. Medical Skin Camouflage: A Recovery Intervention for Female Prisoners who Self-Harm? *Crim Behav Ment Health* 2014;24(5):317-20 doi:10.1002/cbm.1943 [published 03 November 2014].

19 Kenning C, Cooper J, Short V, et al. Prison staff and women prisoner's views on self-harm; their implications for service delivery and development: A qualitative study. *Crim Behav Ment Health* 2010;20(4):274-84 doi:10.1002/cbm.777 [published online 05 July 2010].

20 Ranote S. Skin camouflage service: A first for mental health; Innovation to implementation. Talk presented at Self-harm in Women's Prisons Conference; 2016; University of Manchester.

21 Changing Faces. Skin Camouflage Practitioner Training Trainer's Practical Course Manual for COVER Project. London, England: Changing Faces, 2015.

For peer review only - http://bmjopen.bmj.com/site/about/guidelines.xhtml

For peer review only

22 Walker T, Shaw J, Turpin C, et al. The WORSHIP II study: a pilot of psychodynamic interpersonaltherapy with women offenders who self-harm. *J Forens Psychiatry Psychol* 2017;28(2):158-71 doi:10.1080/14789949.2017.1301529 [published online 08 March 2017].

23 Finlay AY, Khan GK. Dermatology Life Quality Index (DLQI)—a simple practical measure for routine clinical use. *Clin Exp Dermatol* 1994;19:210-6 doi:10.1111/j.1365-2230.1994.tb01167.x [published May 1994].

24 Rosenberg M. Society and the adolescent self-image. Princeton, N.J: Princeton University Press; 1965.

25 Gratz KL. Measurement of Deliberate Self-Harm: Preliminary Data on the Deliberate Self-Harm Inventory. *J Psychopathol Behav Assess* 2001;23(4):253-63 doi: 10.1023/A:1012779403943 [published December 2001].

26 Zanarini MC. Zanarini Rating Scale For Borderline Personality Disorder (ZAN-BPD): A Continuous Measure of DSM-IV Borderline Psychopathology. *J Pers Disord* 2003;17(3):233-242 doi: 10.1521/pedi.17.3.233.22147 [published July 2003].

27 Tennant R, Hiller L, Fishwick R, et al. The Warwick-Edinburgh Mental Well-being Scale (WEMWBS): development and UK validation. *Health Qual Life Outcomes* 2007;5(63) doi:10.1186/1477-7525-5-63 [published 27 November 2007].

28 Beck AT, Kovacs M, Weissman A. Assessment of Suicidal Intention - Scale for Suicide Ideation. *J Consult Clin Psych* 1979;47(2):343-52 doi:10.1037/0022-006X.47.2.343[published April 1979].

29 Beck AT, Steer RA, Brown GK. Manual for the BDI-II. San Antonio: TX: Psychological Corporation; 1996.

30 Wang YP, Gorenstein C. Psychometric properties of the Beck Depression Inventory-II: a comprehensive review. *Rev Bras Psiquiatr* 2013;35:416-31 doi:10.1590/1516-4446-2012-1048 [published online 23 December 2013].

For peer review only - http://bmjopen.bmj.com/site/about/guidelines.xhtml

31 Beck AT, Weissman A, Lester D, et al. The Measurement of Pessimism: The Hopelessness Scale. *J Consult Clin Psych* 1974;42(6):861-5 doi:10.1037/h0037562 [published December 1974].

32 Boduszek D, Shevlin M, Mallett J, et al. Dimensionality and construct validity of the Rosenberg Self-Esteem Scale within a sample of recidivistic prisoners. *J Crim Psychol* 2012;2(1):19-25 doi: 10.1108/20093821211210468.

33 Gusi N, Olivares PR, Rajendram R. The EQ-5D Health-Related Quality of Life Questionnaire. Springer. USA, New York. 2010:87-99.

34 Haywood KL, Garratt AM, Dziedzic K, et al. Generic measures of health-related quality of life in ankylosing spondylitis: reliability, validity and responsiveness. *Rheumatology* 2002;41:1380-87 doi: 10.1093/rheumatology/41.12.1380 [published 01 December 2002].

35 Ware JJ, Kosinski M, Keller SD. A 12-Item Short-Form Health Survey: construction of scales and preliminary tests of reliability and validity. *Med Care* 1996;34(3):220-33 doi:10.2307/3766749 [published April 1996].

36 Barrett B, Byford S. Collecting service use data for economic evaluation in DSPD populations: development of the Secure Facilities Service Use Schedule. *Br J Psychiatry* 2007;190(suppl. 49):s79-s79 doi:10.1192/bjp.190.5.s75 [published online 03 January 2018].

37 Braun V, Clark V. Using Thematic Analysis in Psychology. *Qual Res Psychol* 2006;3(2):77-101 doi:10.1191/1478088706qp063oa [published online 21 July 2008].

38 Boyatzis R. Transforming Qualitative Information: Thematic Analysis and Code Development. California: Sage 1998.

39 Mays N, Pope C. Qualitative research: Rigor and qualitative research. *Brit Med J* 1995;311:109-12 doi:10.1136/bmj.311.6997.109 [published 08 July 1995].

For peer review only - http://bmjopen.bmj.com/site/about/guidelines.xhtml

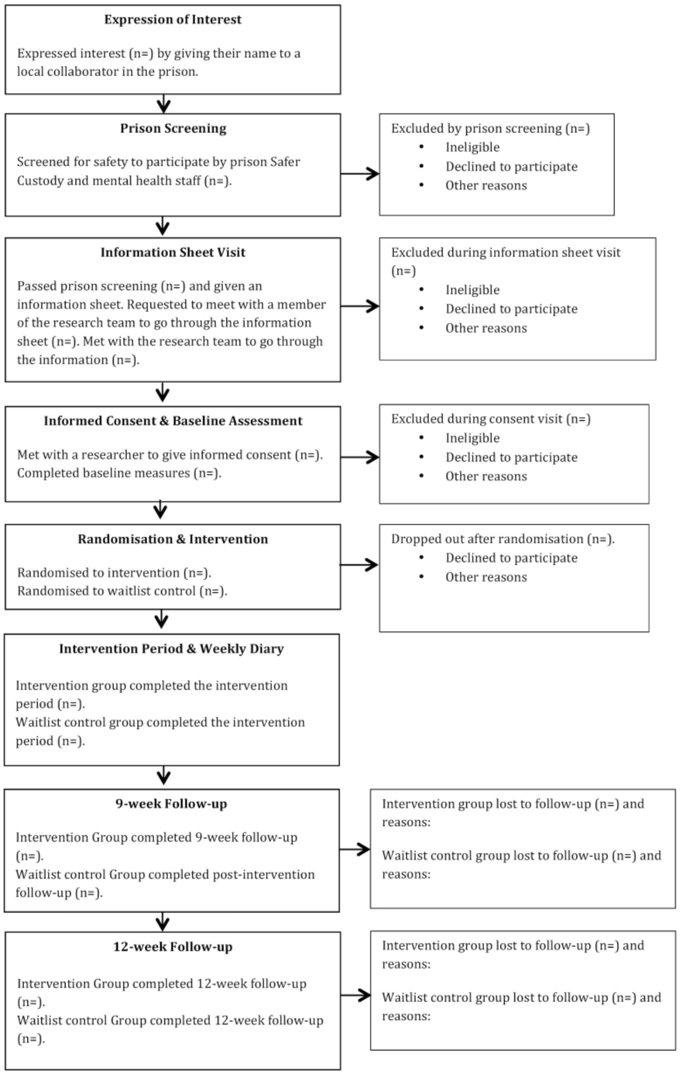

Figure 1. Consort diagram.

239x310mm (600 x 600 DPI)

SPIRIT
STANDARD PROTOCOL ITEMS: RECOMMENDATIONS FOR INTERVENTIONAL TRIALS

SPIRIT 2013 Checklist: Recommended items to address in a clinical trial protocol and related documents*

Section/item	Item No	Description
**Administrative information**		
Title	1	Descriptive title identifying the study design, population, interventions, and, if applicable, trial acronym
Trial registration	2a	Trial identifier and registry name. If not yet registered, name of intended registry
	2b	All items from the World Health Organization Trial Registration Data Set
Protocol version	3	Date and version identifier
Funding	4	Sources and types of financial, material, and other support
Roles and responsibilities	5a	Names, affiliations, and roles of protocol contributors
	5b	Name and contact information for the trial sponsor
	5c	Role of study sponsor and funders, if any, in study design; collection, management, analysis, and interpretation of data; writing of the report; and the decision to submit the report for publication, including whether they will have ultimate authority over any of these activities
	5d	Composition, roles, and responsibilities of the coordinating centre, steering committee, endpoint adjudication committee, data management team, and other individuals or groups overseeing the trial, if applicable (see Item 21a for data monitoring committee)
**Introduction**		
Background and rationale	6a	Description of research question and justification for undertaking the trial, including summary of relevant studies (published and unpublished) examining benefits and harms for each intervention
	6b	Explanation for choice of comparators
Objectives	7	Specific objectives or hypotheses
Trial design	8	Description of trial design including type of trial (eg, parallel group, crossover, factorial, single group), allocation ratio, and framework (eg, superiority, equivalence, noninferiority, exploratory)

For peer review only

Methods: Participants, interventions, and outcomes

Study setting	9	Description of study settings (eg, community clinic, academic hospital) and list of countries where data will be collected. Reference to where list of study sites can be obtained
Eligibility criteria	10	Inclusion and exclusion criteria for participants. If applicable, eligibility criteria for study centres and individuals who will perform the interventions (eg, surgeons, psychotherapists)
Interventions	11a	Interventions for each group with sufficient detail to allow replication, including how and when they will be administered
	11b	Criteria for discontinuing or modifying allocated interventions for a given trial participant (eg, drug dose change in response to harms, participant request, or improving/worsening disease)
	11c	Strategies to improve adherence to intervention protocols, and any procedures for monitoring adherence (eg, drug tablet return, laboratory tests)
	11d	Relevant concomitant care and interventions that are permitted or prohibited during the trial
Outcomes	12	Primary, secondary, and other outcomes, including the specific measurement variable (eg, systolic blood pressure), analysis metric (eg, change from baseline, final value, time to event), method of aggregation (eg, median, proportion), and time point for each outcome. Explanation of the clinical relevance of chosen efficacy and harm outcomes is strongly recommended
Participant timeline	13	Time schedule of enrolment, interventions (including any run-ins and washouts), assessments, and visits for participants. A schematic diagram is highly recommended (see Figure)
Sample size	14	Estimated number of participants needed to achieve study objectives and how it was determined, including clinical and statistical assumptions supporting any sample size calculations
Recruitment	15	Strategies for achieving adequate participant enrolment to reach target sample size

Methods: Assignment of interventions (for controlled trials)

Allocation:

Sequence generation	16a	Method of generating the allocation sequence (eg, computer-generated random numbers), and list of any factors for stratification. To reduce predictability of a random sequence, details of any planned restriction (eg, blocking) should be provided in a separate document that is unavailable to those who enrol participants or assign interventions

Allocation concealment mechanism	16b	Mechanism of implementing the allocation sequence (eg, central telephone; sequentially numbered, opaque, sealed envelopes), describing any steps to conceal the sequence until interventions are assigned
Implementation	16c	Who will generate the allocation sequence, who will enrol participants, and who will assign participants to interventions
Blinding (masking)	17a	Who will be blinded after assignment to interventions (eg, trial participants, care providers, outcome assessors, data analysts), and how
	17b	If blinded, circumstances under which unblinding is permissible, and procedure for revealing a participant's allocated intervention during the trial
**Methods: Data collection, management, and analysis**		
Data collection methods	18a	Plans for assessment and collection of outcome, baseline, and other trial data, including any related processes to promote data quality (eg, duplicate measurements, training of assessors) and a description of study instruments (eg, questionnaires, laboratory tests) along with their reliability and validity, if known. Reference to where data collection forms can be found, if not in the protocol
	18b	Plans to promote participant retention and complete follow-up, including list of any outcome data to be collected for participants who discontinue or deviate from intervention protocols
Data management	19	Plans for data entry, coding, security, and storage, including any related processes to promote data quality (eg, double data entry; range checks for data values). Reference to where details of data management procedures can be found, if not in the protocol
Statistical methods	20a	Statistical methods for analysing primary and secondary outcomes. Reference to where other details of the statistical analysis plan can be found, if not in the protocol
	20b	Methods for any additional analyses (eg, subgroup and adjusted analyses)
	20c	Definition of analysis population relating to protocol non-adherence (eg, as randomised analysis), and any statistical methods to handle missing data (eg, multiple imputation)
**Methods: Monitoring**		
Data monitoring	21a	Composition of data monitoring committee (DMC); summary of its role and reporting structure; statement of whether it is independent from the sponsor and competing interests; and reference to where further details about its charter can be found, if not in the protocol. Alternatively, an explanation of why a DMC is not needed

	21b	Description of any interim analyses and stopping guidelines, including who will have access to these interim results and make the final decision to terminate the trial
Harms	22	Plans for collecting, assessing, reporting, and managing solicited and spontaneously reported adverse events and other unintended effects of trial interventions or trial conduct
Auditing	23	Frequency and procedures for auditing trial conduct, if any, and whether the process will be independent from investigators and the sponsor
**Ethics and dissemination**		
Research ethics approval	24	Plans for seeking research ethics committee/institutional review board (REC/IRB) approval
Protocol amendments	25	Plans for communicating important protocol modifications (eg, changes to eligibility criteria, outcomes, analyses) to relevant parties (eg, investigators, REC/IRBs, trial participants, trial registries, journals, regulators)
Consent or assent	26a	Who will obtain informed consent or assent from potential trial participants or authorised surrogates, and how (see Item 32)
	26b	Additional consent provisions for collection and use of participant data and biological specimens in ancillary studies, if applicable
Confidentiality	27	How personal information about potential and enrolled participants will be collected, shared, and maintained in order to protect confidentiality before, during, and after the trial
Declaration of interests	28	Financial and other competing interests for principal investigators for the overall trial and each study site
Access to data	29	Statement of who will have access to the final trial dataset, and disclosure of contractual agreements that limit such access for investigators
Ancillary and post-trial care	30	Provisions, if any, for ancillary and post-trial care, and for compensation to those who suffer harm from trial participation
Dissemination policy	31a	Plans for investigators and sponsor to communicate trial results to participants, healthcare professionals, the public, and other relevant groups (eg, via publication, reporting in results databases, or other data sharing arrangements), including any publication restrictions
	31b	Authorship eligibility guidelines and any intended use of professional writers
	31c	Plans, if any, for granting public access to the full protocol, participant-level dataset, and statistical code

For peer review only - http://bmjopen.bmj.com/site/about/guidelines.xhtml

Appendices

Informed consent materials	32	Model consent form and other related documentation given to participants and authorised surrogates
Biological specimens	33	Plans for collection, laboratory evaluation, and storage of biological specimens for genetic or molecular analysis in the current trial and for future use in ancillary studies, if applicable

*It is strongly recommended that this checklist be read in conjunction with the SPIRIT 2013 Explanation & Elaboration for important clarification on the items. Amendments to the protocol should be tracked and dated. The SPIRIT checklist is copyrighted by the SPIRIT Group under the Creative Commons "Attribution-NonCommercial-NoDerivs 3.0 Unported" license.

For peer review only

BMJ Open

An acceptability and feasibility pilot randomised controlled trial of medical skin camouflage for recovery of women prisoners with self-harm scarring (COVER): the study protocol.

Journal:	*BMJ Open*
Manuscript ID	bmjopen-2018-021891.R1
Article Type:	Protocol
Date Submitted by the Author:	08-Jun-2018

Complete List of Authors:	Mitchell, Heather; University of Manchester, Centre for Women's Mental Health; Greater Manchester Mental Health NHS Foundation Trust, Psychological Research<br>Abel, Kathryn; University of Manchester, Centre for Women's Mental Health; Greater Manchester Mental Health NHS Foundation Trust, Psychological Research<br>Dunlop, Brendan; University of Manchester, Centre for Women's Mental Health; Greater Manchester Mental Health NHS Foundation Trust, Psychological Research<br>Walker, Tammi; University of Huddersfield, Department of Psychology<br>Ranote, Sandeep; North West Boroughs Healthcare NHS Foundation Trust<br>Robinson, Louise; Lancashire Care NHS Foundation Trust; University of Manchester, Centre for Women's Mental Health<br>Edgar, Fiona; University of Manchester, Centre for Women's Mental Health; University of Central Lancashire, Mental Health and Criminal Justice Group<br>Millington, Tracy; University of Manchester, Centre for Women's Mental Health<br>Meacock, Rachel; Univ Manchester,<br>Shaw, Jennifer; University of Manchester, Offender Health Research Network<br>Gutridge, Kerry; University of Manchester, Centre for Women's Mental Health

**Primary Subject Heading**:	Mental health
Secondary Subject Heading:	Health economics, Legal and forensic medicine, Mental health, Public health
Keywords:	Medical skin camouflage, self-harm, scarring, women prisoners, recovery, feasibility trial

SCHOLARONE™
Manuscripts

An acceptability and feasibility pilot randomised controlled trial of medical skin camouflage for recovery of women prisoners with self-harm scarring (COVER): the study protocol.

Heather Mitchell, [1,2] Professor Kathryn Abel, [1,2] Brendan J Dunlop, [1,2] Dr Tammi Walker, [3] Dr Sandeep Ranote, [4] Dr Louise Robinson, [5,6] Fiona Edgar,[1, 7] Tracy Millington,[1] Rachel Meacock, [8] Professor Jenny Shaw,[9] Dr Kerry Gutridge.[1]

Heather Mitchell, BA
[1]Centre for Women's Mental Health, Division of Psychology and Mental Health, School of Health Sciences, Faculty of Biology, Medicine and Health, University of Manchester, Jean McFarlane Building, Manchester, M13 9PL and [2]Greater Manchester Mental Health NHS Foundation Trust, Bury New Road, Prestwich, M25 3BL, United Kingdom.
heather.mitchell@manchester.ac.uk

Professor Kathryn Abel, PhD, MB ChB
[1]Centre for Women's Mental Health, Division of Psychology and Mental Health, School of Health Sciences, Faculty of Biology, Medicine and Health, University of Manchester, Jean McFarlane Building, Manchester, M13 9PL and [2]Greater Manchester Mental Health NHS Foundation Trust, Bury New Road, Prestwich, M25 3BL, United Kingdom.
kathryn.abel@manchester.ac.uk

Brendan J Dunlop, BSc
[1]Centre for Women's Mental Health, Division of Psychology and Mental Health, School of Health Sciences, Faculty of Biology, Medicine and Health, University of Manchester, Jean McFarlane Building, Manchester, M13 9PL and [2]Greater Manchester Mental Health NHS Foundation Trust, Bury New Road, Prestwich, M25 3BL, United Kingdom.
brendan.dunlop@manchester.ac.uk

Dr Tammi Walker, PhD
[3]Department of Psychology, School of Human & Health Sciences, University of Huddersfield, Edith Key Building, Queensgate, Huddersfield, HD1 3DH, United Kingdom.
t.walker@hud.ac.uk

Dr Sandeep Ranote, MB ChB
[4]5 Boroughs Partnership NHS Foundation Trust, Hollins Park Hospital, Hollins Lane, Winwick, Warrington, WA2 8WA, United Kingdom.

For peer review only
For peer review only - http://bmjopen.bmj.com/site/about/guidelines.xhtml

sandeep.ranote@5bp.nhs.uk

Dr Louise Robinson, MB ChB

[5]Lancashire Care NHS Foundation Trust, Preston, United Kingdom and [6]Centre for Mental Health and Risk, Division of Psychology and Mental Health, School of Health Sciences, Faculty of Biology, Medicine and Health, University of Manchester, Jean McFarlane Building, Manchester, M13 9PL, United Kingdom.

louise.robinson@manchester.ac.uk

Fiona Edgar

[1]Centre for Women's Mental Health, Division of Psychology and Mental Health, School of Health Sciences, Faculty of Biology, Medicine and Health, University of Manchester, Jean McFarlane Building, Manchester, M13 9PL, United Kingdom and [7]Mental Health and Criminal Justice Group, University of Central Lancashire, Preston, United Kingdom .

FEdgar@uclan.ac.uk

Tracy Millington

[1]Centre for Women's Mental Health, Division of Psychology and Mental Health, School of Health Sciences, Faculty of Biology, Medicine and Health, University of Manchester, Jean McFarlane Building, Manchester, M13 9PL, United Kingdom.

7milliebob@gmail.com

Rachel Meacock, MA

[8]Manchester Centre for Health Economics, Institute of Population Health, Division of Population Health, Health Services Research and Primary Care, University of Manchester, Jean McFarlane Building, Manchester, M13 9PL, United Kingdom.

rachel.meacock@manchester.ac.uk

Professor Jenny Shaw, PhD, MB ChB

[9]Offender Health Research Network, Division of Psychology and Mental Health, School of Health Sciences, Faculty of Biology, Medicine and Health, University of Manchester, Jean McFarlane Building, Manchester, M13 9PL, United Kingdom.

jennifer.j.shaw@manchester.ac.uk

Dr Kerry Gutridge, PhD (corresponding author)

For peer review only - http://bmjopen.bmj.com/site/about/guidelines.xhtml

[1]Centre for Women's Mental Health, Division of Psychology and Mental Health, School of Health Sciences, Faculty of Biology, Medicine and Health, University of Manchester, Jean McFarlane Building, Manchester, M13 9PL, United Kingdom.
kerry.gutridge@manchester.ac.uk

Word count: 5,342

Abstract

Introduction

Self-harm in prison is a major public health concern. Less than 5% of UK prisoners are women, but they carry out more than a fifth of prison self-harm. Scars resulting from self-harm can be traumatising and stigmatising, yet there has been little focus on recovery of women prisoners with self-harm scarring. Medical skin camouflage (MSC) clinics treat individuals with disfiguring skin conditions, with evidence of improved wellbeing, self-esteem and social interactions. Only one community study has piloted the use of MSC for self-harm scarring.

Methods & Analysis

We describe an acceptability and feasibility pilot randomised controlled trial; the first to examine MSC for women prisoners who self-harm. We aim to randomise 20-25 women prisoners to a 6-week MSC intervention and 20-25 to a wait-list control (to receive the MSC after the study period). We aim to train at least 6-10 long-term prisoners with personal experience of self-harm to deliver the intervention. Pre- and post-intervention, we will pilot collection of women-centred outcomes, including quality of life, wellbeing and self-esteem. We will pilot collection of self-harm incidents during the intervention, resources used to manage/treat self-harm and follow-up of women at 12-weeks from baseline. Data on recruitment, retention and drop-out will be recorded. We aim for the acceptability of the intervention to prison staff and women prisoners to be explored in qualitative interviews and focus groups.

Ethics and dissemination

For peer review only

Ethical approval for COVER has been granted by the North East – York REC for Phase 1 and 2 (REC reference: 16/NE/0030) and West of Scotland REC 3 for Phase 3 and 4 (REF: 16/WS/0155). Informed consent will be the primary consideration; it will be made clear that participation will have no effect on life in prison or eligibility for parole. Due to the nature of the study, disclosures of serious self-harm may need to be reported to prison officials. We aim for findings to be disseminated via events at the study prison, presentations at national/international conferences, journal publications, prison governor meetings and university/NHS trust communications.

Trial Registration

The trial is registered on clinicaltrials.gov (identifier: NCT02638974).

Strengths & limitations of this study

- COVER is the first pilot randomised controlled trial of the use of medical skin camouflage for women who self-harm in prison
- The study has been co-designed with experts-by-experience to test the delivery of a peer-led intervention
- As a pilot, the sample size for the study is small, however, the research is designed to gather data on the feasibility and acceptability of delivering the intervention in prison rather than the efficacy of the intervention
- The study will take place in one prison within the women's estate.

Key Words: Medical skin camouflage, self-harm, scarring, women prisoners, recovery, feasibility trial.

INTRODUCTION

Self-harm in Women's Prisons

Self-harm is defined as 'intentional self-poisoning or injury, irrespective of the apparent purpose of the act'[1]. The most common methods for self-harm in women's prisons are cutting and scratching

For peer review only - http://bmjopen.bmj.com/site/about/guidelines.xhtml

For peer review only

followed by self-strangulation[2]. This complex behaviour is an increasing public health concern, not least because of its association with acute psychological distress and increased suicide risk[2, 3].

Self-harm is extremely prevalent and increasing in UK prisons. In the 12 months to December 2016 there were 7,657 incidents of self-harm in female prisons, an increase of 4% on the previous year[4]. This is a rate of 1,987 self-harm incidents per 1,000 prisoners. Although women make up approximately 5% of the UK prison population, they are responsible for around a fifth of all prison self-harm[4].

Research has shown that living with disfigurement from non-self-harm causes can have long-term physical and psychosocial effects, including reduced social interaction, increased social anxiety and reduced quality of life[5, 6]. Furthermore, living with scars can be challenging in a society which values physical attractiveness[7, 8]. It is likely that women prisoners with self-harm scarring experience similar psychosocial difficulties e.g. low self-esteem and interpersonal problems. These may be exacerbated by guilt and shame that women may feel because of their self-inflicted injuries[9]. There are, however, individuals who feel ambivalent about their self-harm scars, and whilst they may attempt to conceal scars in certain contexts, some feel confident and comfortable with their physical appearance[10].

Medical Skin Camouflage

Medical skin camouflage (MSC) uses British National Formulary-listed preparations to reduce the visibility of scarring or disfigurement[11], with the potential to restore self-esteem, and aid recovery[12, 13, 14]. Products include skin-matched creams and powders that are waterproof, opaque and allow adherence to textured skin. All the products are 'borderline prescription' products that are available on NHS prescription at each prescriber's discretion. A systematic review of the use of MSC in prisons yielded no available studies. Only a handful of published studies have evaluated the emotional/psychological benefits of MSC and all were in dermatological diseases or burns scarring. They report significant psychological benefit, improved social and sexual relationships and improved

For peer review only

employability[8, 15, 16]. Despite these potential benefits, few services offer MSC for self-harm scarring[17].

There has been little focus on how prisoners feel about their self-harm scars and no formally evaluated interventions to help women cope with any related psychosocial difficulties. This is the first study to formally deliver and evaluate an MSC intervention in a women's prison. Potential benefits of the intervention may include 1) increased self-esteem, confidence and quality of life; 2) empowering women to take part in work and social activities they might otherwise avoid and 3) enhancing the strategies and interventions that prison staff have to work with self-harm[18]. Previous work by the research team has shown that there is a difficult relationship between prison staff and prisoners who self-harm and that staffs feel restricted in how to help women[19]. This intervention may help staff to support women with self-harm scars and promote positive staff attitudes about self-harm and its management.

This study has been developed in collaboration with staff from 5 Boroughs Partnership (5BP) NHS Foundation Trust who recently piloted an innovative camouflage service for service users with self-harm scars[20]. The 6-month pilot found that 95% of young people who used the MSC experienced improved confidence and ability to engage in activities[20]. To our knowledge, this is the first time that MSC has been evaluated in a mental health service and provided as part of a recovery package. The 5BP MSC service continues to be run in partnership with Changing Faces, a registered charity that uses volunteers to teach the MSC techniques to people in the community. This feasibility and acceptability study would provide insight into any benefits of using MSC in women's prisons and also any downsides, risks or unintended consequences.

Phase 1 and 2

The MSC intervention and protocol used in our study were informed by the Changing Faces MSC training materials[21] and modified in Phase 1 and 2 of the project. Phase 1 involved one focus group, with women prisoners with experience of self-harm (n=10) and one with prison staff (n=10). Both

For peer review only - http://bmjopen.bmj.com/site/about/guidelines.xhtml

groups were conducted in safer custody meeting rooms and lasted between 60-90 minutes. The staff focus group explored and refined practical aspects of delivering MSC in the prison, including details of how participants would be recruited, where MSC clinics would be held and whether any MSC items would be unsuitable for prison use. The focus group with women prisoners helped to select the set of women-centred outcome measures and discussed their thoughts on long-term prisoners delivering the intervention. Women said they would prefer to be trained by other prisoners, particularly other women who have self-harmed. The rationale for recruiting long-term prisoners to deliver the intervention was to improve the sustainability of the intervention since they are likely to remain in the prison for a long time and can therefore continue training women to use MSC. Women also discussed the idea of completing a weekly diary; they thought this would be a good way of recording any thoughts or incidents of self-harm and some women had used a diary previously. Phase 2 involved adapting the MSC treatment intervention based on these focus groups, and producing the training and intervention protocols. The full analysis of the focus groups will be reported in a separate paper.

Study Aims:

1) To evaluate the feasibility and acceptability of a Randomised Controlled Trial (RCT) of MSC for women prisoners with self-harm scarring.
2) To assess the feasibility and acceptability of long-term prisoners delivering the MSC intervention.
3) To test the feasibility and acceptability of collecting a set of women-centred outcome measures pre- and post-intervention, as well as a weekly self-harm diary.
4) To pilot follow-up of women at 12 weeks after baseline.
5) To test the feasibility and acceptability of collecting resource use data relating to self-harm incidents.

METHODS & ANALYSIS

For peer review only - http://bmjopen.bmj.com/site/about/guidelines.xhtml

Design

This study is a feasibility pilot of an RCT, incorporating a qualitative component to assess the acceptability of MSC to women prisoners and prison staff. The study is taking place in one UK closed women's prison. The research is funded by the National Institute for Health Research (NIHR) Research for Patient Benefit Programme (PB-PG-1013-32075). It was approved by North East – York REC for Phase 1 and 2 (REC reference: 16/NE/0030) and West of Scotland REC for Phase 3 and 4 (16/WS/0155). The current protocol version is Version 6 (17/05/2017).

Patient and public involvement

At the development phase of the research, a patient and public involvement group was conducted in one women's prison using a Patient and Public Involvement bursary from the NIHR Research Design Service North West. During this group women from the prison, who had self-harm scarring, contributed towards the research topic development through discussion of the possible impact of medical skin camouflage. This informed the outcome measures for the research and the topic guides/interview schedules for the qualitative work. In phase 1 of the research, women in prison with self-harm scarring, helped refine the design of the research assessing the burden of involvement in a randomised controlled trial.

Two experts-by-experience joined the research team at the start of the research and contributed towards the design of all the materials for participants. In phase 3, one of these experts-by-experience will help train the long-term prisoners to be medical skin camouflage practitioners having agreed to allow the women to practice application of the MSC on her self-harm scars. Another of our experts-by-experience, who is a trained qualitative researcher, analysed the phase 1 focus group data and will co-facilitate the staff focus group at the end of the research. A current prisoner, who works in Safer Custody has agreed to help to organise the participants' appointments in the prison and will sit on the project steering group. At the end of the research our experts-by-experience will help us to design a dissemination event for the women in prison that will involve presentations on the research outcomes.

For peer review only - http://bmjopen.bmj.com/site/about/guidelines.xhtml

A plain English summary of the research will also be provided to women in the prison. We will also disseminate the research on the closed prison radio system.

Sample size

Over 6 months (January 2017 – May 2017), we aim to recruit at least 6-10 long-term women prisoners to be trained in MSC. These women will then deliver the intervention to trial participants. The long-term prisoners will not be participants in the RCT, but will instead form an integral part of the research team delivering the intervention to the RCT participants.

Over seventeen months (January 2017 – May 2018) we aim to recruit and consent 40-50 women prisoners to be randomised to receive either MSC or 'treatment as usual' (TAU) in a wait-list control (to receive the MSC after the study period) design. Based on previous research[22] and recent figures from the study prison, we estimate that there will be around 5-6 eligible women per month. The sample size is based on a prediction that approximately half of these eligible women will be interested in the research.

Participants and recruitment procedures

Recruitment procedures and advertisement strategies have been informed by the Phase 1 focus groups. The research team will advertise the research at Safer Custody meetings attended by women prisoners. Leaflets and posters will be distributed to our local collaborators in the prison for display in different locations around the prison. Women will inform the local collaborators if they are interested in participating. We have one member of Safer Custody staff collaborating with the researchers and a woman prisoner from the Safer Custody team organising the research appointments. Prison staff will assess all volunteers to determine whether they would pose a risk to the researchers or other participants. Staff will also check the woman's sentence length to ensure she has enough time remaining on her sentence to take part in the study. In addition, healthcare staff will review the list of women to assess whether there are any health reasons why they might not be safe to participate. This has been usual practice across our decade of prisoner participation in research.

For peer review only - http://bmjopen.bmj.com/site/about/guidelines.xhtml

Vetted/screened women will be provided with an information sheet and offered the opportunity for the research team to visit, read through the sheet and answer any questions. Consent for the research will be agreed at least 24 hours after the information sheet has been read.

Inclusion/exclusion criteria

Phase 3:

We aim to recruit 6-10 long-term prisoners with at least 10 years or more left on their sentence and who have experience of self-harm.

Discussions with prison staff suggested that the most suitable long-term women would be those who already hold a position of responsibility in the prison e.g. a peer supporter or trained Samaritan listener.

Phase 4:

We aim to recruit 40-50 women prisoners screened for date of release, with sufficient time left on their sentence to complete the intervention period. The women will have self-harm scarring anywhere on their body that they are happy to show to others, with at least some closed wounds (to allow the MSC to be applied).

All participants (phase 3 and 4) will be aged 18 or older and able to give written, informed consent. Capacity to consent will be assessed by the experienced researchers (HM and KG) in collaboration with Safer Custody and Mental Health Care contacts in the prison. Participants will be excluded from the study if they are unable to provide written informed consent, or if they pose a risk to researchers (as assessed by the prison).

Randomisation

In Phase 4 internet randomisation (using an internet-based programme to randomise participants; www.sealedenvelope.com) will be carried out by the non-blind members of the research team, KG or HM, to allocate eligible women to MSC or waitlist control. Women in the waitlist group would

For peer review only

For peer review only - http://bmjopen.bmj.com/site/about/guidelines.xhtml

receive one skin-matched prescription of MSC at the end of the research. Waitlist control has been chosen as the comparator to give all participants an opportunity to use the MSC. Participants randomised to the waitlist control would be aware of their allocation (figure 1).

The Intervention – Medical Skin Camouflage for Self-harm scarring (adapted for delivery in a women's prison)

Development

Outcomes of the Phase 1 focus groups informed the development of the MSC intervention materials. In addition, two service user researchers (FE and TM) provided guidance to the research team, focusing on whether the intervention materials were suitable in terms of readability and sensitivity.

The intervention package consists of the training manual and four additional documents. The main training manual has been adapted from training manuals used by Changing Faces[21]. The adapted materials have been reviewed by a representative from the charity, to ensure that all key learning and safety points are covered.

Manual Content

The 34-page training manual has 13 sections that are listed and briefly described in Table 1 below.

Table 1: Sections of the training manual

Section Number	Section Name	Overview	Key Learning Point
1	Self-harm	This section aims to help women understand the different forms that self-harm can take and different reasons why women self-harm.	Different people have very different reasons for self-harming and it is therefore important to not make assumptions.
2	Working with women who self-harm in the COVER project	This section covers how to manage confidentiality and how to work with women who self-harm e.g. being respectful, don't judge the participant, the limits of confidentiality.	To manage and understand the limits of confidentiality, e.g. if she discloses something that puts her or someone else at risk, and what to do if a woman becomes upset.

For peer review only - http://bmjopen.bmj.com/site/about/guidelines.xhtml

3	Hygiene	This section covers how to run a hygienic skin camouflage clinic and how to keep the kit clean.	Hygiene rules to follow during an appointment.
4	Communication	This section covers communication rules, including how to manage participant expectations e.g. setting realistic expectations for what MSC can achieve.	Understanding the importance of helping the client to express their wishes and working with them to achieve the best results.
5	The Skin & Skin types	An overview of preparing the skin for application of MSC and how to ensure safe usage e.g. by checking for allergies.	How to prepare the skin and when it is not safe to use the products.
6	Overview of the kit	This section describes the items in the MSC kits and how to lay them out in a logical order.	Laying the kit out in a logical order will help the practitioner to quickly identify the products.
7	Colour Matching	This section covers colour matching. This will involve some practical activities on identifying colour tones and colour matching.	To be able to identify tones in the creams and perform a colour match.
8	Brush Technique	An overview of the brush technique and when/how to use it.	To understand when and how to use brushes.
9	Finger Technique	An overview of the finger technique and when/how to use it.	To understand when and how to use the finger technique.
10	Sponge Technique	An overview of the sponge technique and when/how to use it.	To understand when and how to use sponges.
11	Spreading Technique	An overview of the spreading technique and when/how to use it.	To understand when and how to use the spreading technique.
12	Working with powder	An overview of how to use powder to set the MSC creams.	To understand the purpose of powder, and how to apply it.
13	Completing the record card	This section covers how to complete the participant record card, including what to do with the record card after the appointment.	What to include on the record card.

Accompanying Documents

1) A single sheet of key learning points for long-term prisoners covering safety issues such as how to protect trial participants e.g. breaking confidentiality if a woman discloses something which suggests she or someone else is at risk of harm.

2) A monitoring sheet for long-term prisoners to be used in weekly meetings with the research team. The form will help to identify whether any further training or support is required.

For peer review only - http://bmjopen.bmj.com/site/about/guidelines.xhtml

3) An appointment checklist for long-term prisoners breaking down the 14 core steps in a MSC appointment, from laying out the kit, to completing a prescription record card.
4) A DOs and DON'Ts sheet for trial participants: this covers reminder points, including those related to safety and hygiene (e.g. always keep lids on the products) and some rules relating to continued participation in the trial (e.g. don't trade or share the products as only one prescription will be provided, added at the request of prison staff).

Delivery of the Intervention

Three stages of delivery: 1) training sessions for long-term prisoners, 2) skin camouflage clinics run by long-term prisoners for trial participants, 3) prescription of MSC products by prison healthcare.

1) Members of the research team aim to deliver a half-day group training session to 6-10 long-term women prisoners. During this session, the research team will work through the training manual, answering any questions and giving practical demonstrations of colour matching, application techniques and powdering. Participants will participate in practical activities to ensure that they have understood the training and are competent in MSC. There is scope for the training time to be extended if the women require more practice.

2) The aim is that regular skin camouflage appointments will be run by the trained long-term prisoners. The appointments will be held during the core prison day and will not interfere with the women's income. All participants will be seen individually for one hour; the intervention group will be seen as soon as possible after they have been randomised and the waitlist control group will be seen after they have completed their 12-week follow-up. During this appointment, the long-term prisoner will provide the woman with information about the MSC creams and powders; including allergy checks to ensure the woman can safely use the products. The long-term prisoner will then perform a colour-match for the participant and demonstrate the application techniques. The participant will then practice applying the camouflage creams themselves until they are happy with the results. The long-term prisoner will then complete a record form to be given to healthcare.

For peer review only - http://bmjopen.bmj.com/site/about/guidelines.xhtml

3) The aim is for a nurse prescriber from healthcare to meet with all participants (the intervention group at the start and the waitlist control group at the end of the research) and write a prescription for 1x camouflage cream and 1x camouflage powder. Women will be informed that they will only be given one prescription for the duration of the study. The amount of camouflage cream required will depend on the extent of the participant's scarring, but based on the 5BP pilot[20] we anticipate that one prescription will be enough for a 3-month period.

Continued provision of the MSC products post-trial is not envisaged at this stage within the study prison. However, all participants will be given a letter that they have the option to give to their General Practitioner (GP) in the community that will detail their MSC prescription, and will recommend that the product is prescribed to them.

Assessing feasibility and acceptability

We will assess the feasibility of recruiting and randomising women to MSC vs wait list and of long-term prisoners delivering MSC appointments. We will examine use of the MSC, attrition (number of drop-outs at each time point), and retention (the proportion of participants who complete the intervention period). The feasibility of delivery in a prison setting (i.e. location, duration of training, peer-delivery) and the acceptability of the intervention to women and staff will be assessed using qualitative interviews and focus groups. The feasibility of undertaking a full-scale RCT of MSC for women in prison will be assessed by studying recruitment (the proportion of eligible participants consenting to join the study) and completeness of outcome measures at baseline, post-intervention (approximately nine weeks from baseline to include the time taken to receive the MSC and 6 weeks of MSC use) and at follow-up (12 weeks from baseline). We have included a 12-week follow up to assess retention and attrition over a longer period of time. Data will be collected on reasons for ineligibility, non-consent and dropout, including when the participant dropped out/withdrew from the study.

Outcome measures for future RCT design

For peer review only - http://bmjopen.bmj.com/site/about/guidelines.xhtml

For peer review only

The aim is that all participants in both groups (MSC and waitlist control) will be asked to complete a set of quantitative outcome measures at baseline (zero weeks), post-intervention (approximately nine weeks later) and at follow-up (approximately 12 weeks from baseline). This will help us to assess the feasibility and acceptability of these measures for a future clinical and cost-effectiveness RCT. Outcome measures will be administered by the project manager (PM), trained research assistant (RA) or research nurses from the NIHR clinical research network. The PM and the RA will be unblinded to the randomisation outcome and will therefore only administer baseline measures; administration of measures at any other time point by these individuals may bias results. The research nurses will be blinded and will complete the post-intervention and 12-week follow-up assessments. All research assessments (which we anticipate will last approximately 1 hour) will take place in a private room in Safer Custody. The PM, RA or research nurse will complete a case report form for each participant; recording any additional notes on each participant e.g. reasons for questionnaire non-completion. Given the sensitive nature of some of the selected outcome measures, we have consulted with women, Safer Custody staff and healthcare/mental health staff to develop procedures to protect and support participants. If, at any point during a research assessment the woman becomes agitated or distressed, we will ask them if they would like to take a break or if they want to resume the assessment on another day. If the researcher has any concerns for the woman, they will alert the local collaborator who will ensure it is dealt with accordingly using existing prison support systems. The participant information sheet outlines that the researcher is obligated to inform the prison if there is a risk to the participant's health, safety or wellbeing. For this study this will include reporting high suicidal ideation and high risk of serious self-harm.

We aim to administer a selection of outcome measures (see Table 2) to all participants at baseline, 9-weeks and 12-weeks after baseline. Two of these measures, the Dermatology Quality of Life Index (DQLi)[23] and Rosenberg Self-esteem Scale (RSES)[24], were added following focus group discussions on the psychological and interpersonal impact of scars. At baseline, we also aim to use a bespoke demographic and personal history questionnaire to collect relevant personal information including age, ethnicity, whether they are on remand or sentenced, past experience of contact

For peer review only - http://bmjopen.bmj.com/site/about/guidelines.xhtml

psychiatric services, drug dependence and experiences of domestic violence, sexual abuse and parental neglect. We aim to collect this information to check whether the two randomised groups have similar backgrounds. With women's permission, our local collaborator or a research nurse will access information on key forensic and clinical characteristics from CNomis, SystemOne (the prison electronic medical records) and from Assessment, Care in Custody and Teamwork (ACCT) documentation; these systems will be accessed by prison staff unless the researchers are granted access permission. Forensic characteristics will include types of offence (violent or non-violent), sentence length and stage of sentence and clinical characteristics will include psychiatric diagnosis and history. We aim to administer the Deliberate Self-Harm Inventory (DSHI)[25] at baseline and follow-ups: a 17-item questionnaire that assesses the history and frequency of self-harming behaviours. We also aim to administer the Zanarini Rating Scale for Borderline Personality disorder (ZAN-BPD) at all time points as a measure of borderline psychopathology[26].

Table 2: Participant Assessment Schedule

Assessment Tool	Brief Description	Time Point			
		Duration (min)	Baseline	Post-intervention	Follow-up (12 weeks)
Personal History Questionnaire	Socio-demographic/life history	5	X		
DSHI	Methods/history of self-harm	10	X	X	X
WEMWBS	Mental wellbeing	5	X	X	X
BSSI	Suicidal ideation	10	X	X	X
BDI-II	Depression	10	X	X	X
BHS	Hopelessness	5	X	X	X
DQLi	Self-harm scarring quality of life	5	X	X	X
RSES	Self-esteem	5	X	X	X
ZAN-BPD	Borderline personality disorder	5	X	X	X
EQ-5D-5L	Generic health	5	X	X	X
SF12	Generic health/quality of life	5	X	X	X
Qualitative Interview	Acceptability and feasibility	30			X
Total time burden			70	65	95
Self-harm Diary	Self-harm thoughts and incidents	Weekly from baseline to 12 weeks			

We aim to examine whether the Warwick-Edinburgh Mental Well-Being Scale (WEMWBS)[27] is a suitable primary outcome for a full-scale RCT. The WEMWBS is a 14-item scale of mental wellbeing

For peer review only - http://bmjopen.bmj.com/site/about/guidelines.xhtml

covering subjective wellbeing and psychological functioning, in which all items are worded positively and address aspects of positive mental health. The WEMWBS has high internal consistency (α = .91) and test-retest reliability (0.83)[27]. This measure would be used to calculate study power in a full-scale subsequent trial.

Becks Scale for Suicidal Ideation (BSSI)[28]: a 19-item instrument measuring intensity, duration and specificity of thoughts about committing suicide. The BSSI has high internal consistency (0.89) and high inter-rater reliability (0.83)[28]. The BSSI has been successfully used in a pilot trial of Psychodynamic Interpersonal Therapy for women prisoners who self-harm[22].

Becks Depression Inventory (BDI-II)[29]: a 21-item scale measuring symptoms of depression. The BDI-II has high internal consistency and a test-retest reliability ranging from 0.73 to 0.96[30].

Beck Hopelessness Scale (BHS)[31]: a 20-item self-report inventory designed to measure three major aspects of hopelessness: feelings about future, loss of motivation and expectations. The BHS has high concurrent validity (0.86) and high reliability (α = 0.91)[31].

Prison-adapted Dermatology Quality of Life Index (DQLi)[23]: a 7-item questionnaire adapted from a validated 10 item scale that has been used in over 40 different skin conditions in over 80 countries. Test-retest reliability has been found to be high (0.99)[23].

Rosenberg Self-Esteem Scale (RSES)[24]: a 10-item Likert scale with items answered on a four-point scale – from strongly agree to strongly disagree. The scale measures self-esteem and has been used in prison research[32]. Internal consistency ranges from 0.77 to 0.88 and test-retest reliability ranges from 0.82 to 0.85[24].

For peer review only - http://bmjopen.bmj.com/site/about/guidelines.xhtml

For peer review only

EQ-5D-5L[33]: a generic preference-based measure covering five domains of health-related quality of life (mobility, self-care, usual activities, pain/discomfort, anxiety/depression). Test-retest reliability is high and ranges from 0.78 to 0.87, with convergent validity at 0.64[34].

SF-12 is a shortened version of the SF-36[35], consisting of twelve questions covering eight dimensions of health: physical functioning, role limitations - physical, bodily pain, general health, vitality, social functioning, role limitations - emotional, and mental health. Test-retest reliability ranges from 0.76 to 0.89 and relative validity ranges from 0.43 to 0.93 [34].

To reduce attrition, we aim to seek consent at baseline for women who have been transferred or have left prison during the study period to be followed up in person at other prisons or in a public place in the community, following a lone worker policy.

In addition to the outcome measures listed above, we also aim to ask trial participants to complete a weekly diary every week from their baseline assessment. Prison staff and women prisoners in the Phase 1 focus groups proposed the use of a weekly diary; some of the women had completed a diary of self-harm thoughts and events in the past and found it helpful. The research team will collect the diary each week. The diary will ask questions about any thoughts or acts of self-harm that have occurred during the week and any life events that have impacted on their self-harm during the week. Women will also have a free-text space to add additional comments.

We also aim to pilot the collection of resource use data so that we can determine if it is feasible to gather this data in a larger trial, with a view to calculating the cost of treatment in comparison to usual care. This will be collected using the Secure Facilities Service Use Schedule (SFSUS)[36] and a bespoke resource use questionnaire. Resource use data is likely to be extracted by the local collaborator from systems such as CNomis and Officers logs. Prison staff will redact any confidential information. We also aim to use these systems, together with SystemOne, to extract data on self-harm incidents that occurred during the intervention. If we successfully extract the data we will then

For peer review only - http://bmjopen.bmj.com/site/about/guidelines.xhtml

triangulate prison records of self-harm incidents with women's self-reported incidents. We will record the time taken by prison staff and healthcare staff to extract this information.

To inform a future cost analysis, we also aim to record the time spent by Changing Faces training the researchers in medical skin camouflage, time spent by the research team training long-term prisoners to become skin camouflage practitioners, time spent by long-term prisoners delivering the intervention, and quantities of MSC products prescribed.

Qualitative Data

We aim to conduct interviews with all women in the MSC group (n=20) at the end of the study, to assess the acceptability of the intervention to service users. The interviews with women will explore their views on applying MSC, how long it stays on for, how useful they found it and any positive or negative effects on their everyday life, mood, self-esteem and self-confidence. The topic guides have been developed in consultation with two service user researchers and informed by outcomes of the Phase 1 focus group.

We also aim to interview the long-term prisoners to assess their experiences of being an MSC practitioner, in terms of the acceptability of the training, mentoring/support from the research team and any benefits or difficulties working with participants.

In addition, we aim to conduct a focus group with prison staff from different disciplines (including Safer Custody staff, prison officers and healthcare staff) that have been in contact with women involved in the trial. The focus group would explore acceptability of the intervention from a staff perspective, including what they thought about prisoner-delivery of the MSC intervention and whether the intervention has had a positive, or negative, impact on their job or their relationships with women prisoners. All interviews and the focus group will use semi-structured topic guides with open-ended questions that should enable us to explore in-depth the aspects of the intervention that worked well, the aspects that did not work well, and things that could be improved. With permission from

For peer review only - http://bmjopen.bmj.com/site/about/guidelines.xhtml

participants, interviews will be audio-recorded. All recordings will then be transcribed verbatim and analysed using thematic analysis[37].

We aim to assess fidelity to the MSC intervention by a) observing the long-term prisoners at the end of training covering one of our service user researcher's scars; b) audio recording 10% of the training sessions which will be rated for fidelity to the training manual by an independent researcher.

Data Analysis

Quantitative analyses

We shall compare means before and after treatment using descriptive statistics, including standard deviations and confidence intervals for outcome variables to inform sample size estimates for a future RCT. We will also present descriptive statistics on recruitment and retention of participants in both groups, including reasons for dropout at different stages.

We shall assess the feasibility and relevance of both the EQ-5D-5L and SF-12 for the prison population through correlation between changes from baseline to follow-up of these and other piloted measures (WEMWS; BSSI; BDI; BHS; RSES; ZAN-BPD); and examination of completion rates. Descriptive analysis of Health Related Quality of Life data will also inform the suitability of the measures for future clinical and economic evaluations of the intervention.

Resource use collection will also be assessed through time taken to complete questionnaires, completion rates and ability to obtain included resource-use categories to inform suitability of resource use categories in a future economic evaluation. Descriptive analysis of resource use data will also inform future trial design.

Qualitative data

Qualitative data will be analysed using thematic analysis[37]; analysis which will be conducted by the RA and PM and checked for accuracy by an independent researcher. Preliminary codes and categories

For peer review only - http://bmjopen.bmj.com/site/about/guidelines.xhtml

are assigned to the text[38] and emergent themes subject to constant comparison and examined for goodness-of-fit until a final set of key themes identified[39]. Adopting an inductive, iterative approach, data analysis will commence with the first interview.

Data Entry & Storage

Written consent forms and completed questionnaires will be removed straight to the University of Manchester. Participants will be given a unique participant number that will be used on questionnaires and the electronic database. A password-protected document will link participant names and numbers. Any identifying personal data (e.g. consent forms) will be stored separately from other research data. In the University of Manchester this will mean storage in the locked limited access corridor. Electronic databases will be stored on an encrypted space on University of Manchester computers. The RA would enter all data and the PM will carry out 10% checks for accuracy.

ETHICS & DISSEMINATION

Ethical approval for COVER was granted by the North East – York REC for Phase 1 and 2 (REC reference: 16/NE/0030) and West of Scotland REC 3 for Phase 3 and 4 (REF: 16/WS/0155).

Adverse events

All participants will be women who have a history of self-harm. Therefore, self-harm incidents are an expected event and not necessarily a serious adverse event. All adverse events, including incidents of self-harm, will be recorded and reported to the project manager. In consultation with prison staff and the prisoner, the research team will assess the seriousness of the adverse event and whether it is related to project participation; events that are judged as serious and unrelated will be reported to the sponsor only. Events judged as serious and related to project participation will be reported to the research sponsor, host NHS trust and West of Scotland Research Ethics Committee (REC).

Dissemination

We aim for our findings to be disseminated to prisoners, prison staff and to the wider stakeholder (academic and clinical) community via showcase events at the study prison, presentations at national

For peer review only - http://bmjopen.bmj.com/site/about/guidelines.xhtml

and international conferences, journal publications, safer custody and prison governor meetings and university/NHS trust communications. During dissemination, we will hold discussions with key personnel from NHS England and HMPPS regarding future provision of the intervention.

Discussion

Despite the large number of women in prison whom self-harm (or who have self-harmed in the past and are living with scarring), there are little/no evidence-based interventions which aim to improve self-esteem, confidence and wellbeing. This low-cost intervention has the potential. to improve women's mood and how they feel about themselves.

Our Phase 1 focus groups suggested that many women prisoners who repeat self-harm struggle on a regular basis with negative feelings about their scars e.g. they have to cover them in front of others/ family for fear of being judged adversely or upsetting them; they are a constant reminder of bad times or they lack confidence in their bodies because of scars. A prisoner-delivered MSC intervention could reduce such distress women prisoners experience and help them re-integrate into the community without the additional burden of being judged because of their scars.

This intervention was implemented successfully in a community mental health service. We, therefore, anticipate that, with the support of prison staff and long-term prisoners, COVER will provide a beneficial resource to improve wellbeing in an often-neglected population.

Engaging long-term prisoners in the delivery of MSC clinics should increase the sustainability of the intervention if it were to be commissioned in future and provide meaningful work for women prisoners, offering a valuable opportunity to improve relationships between prisoners and contribute towards a therapeutic community with the prison. Peer support schemes, such as the Samaritan's Listener scheme which runs across many UK prisons are increasingly popular, enabling prisoners to develop a range of transferable skills and reducing the burden of distress and self-harm management for prison staff. If successfully implemented, COVER will run alongside these peer support services and provide additional help for women who self-harm.

For peer review only - http://bmjopen.bmj.com/site/about/guidelines.xhtml

Authors' contributions

KA and TW conceived and designed the study and applied for funding. KG drafted the original protocol. KA, KG & HM led the development of the prison-modified MSC intervention; are responsible for drafting and revising the protocol manuscript; have given final approval for the version to be published and are accountable for all aspects of the work. HM and BD led the write-up of the protocol manuscript under the supervision of KG. KG is the research project manager and HM/BD are the research assistants on the study. KA, KG, HM, TW, SR, LR, JS & RM co-led the development of COVER and participated in the design of the study. KA and JS provide the senior academic oversight on all aspects of the feasibility study. KG, HM and BD lead the Patient and Public Involvement (PPI). FE and TM have provided expert by experience input throughout the project. All authors read and approved the final protocol manuscript.

Acknowledgements

We would like to thank the service users, prison staff and healthcare professionals who participated in the focus groups during phase one of the study. We would like to thank Fiona Edgar and Tracey Millington, our experts-by-experience for their valuable insights and contributions and the COVER project steering group for their on-going input and support. We would also like to thank the prison governor and our local collaborators in the prison for their support.

Funding

This project was funded by the National Institute for Health Research (NIHR) Research for Patient Benefit Programme (reference PB-PG-1013-32075). This article presents independent research funded by the NIHR. The views and opinions expressed therein are those of the authors and do not necessarily reflect those of the RfPB programme, NIHR, NHS or the Department of Health.

For peer review only - http://bmjopen.bmj.com/site/about/guidelines.xhtml

The study sponsor is The University of Manchester and the host NHS Trust is Greater Manchester Mental Health NHS Foundation Trust.

Competing Interests

The authors declare that they have no competing interests.

Consent for Publication

Not applicable.

Ethical Approval and Consent to Participate

Ethical approval has been granted by the West of Scotland Research Ethics Committee 3, REC reference: 16/WS/0155. Recruitment is currently on-going; to date, 30 participants have entered the Phase 4 trial.

Availability of Data and Materials

Not applicable.

Author Details

[1]Centre for Women's Mental Health, Division of Psychology and Mental Health, School of Health Sciences, Faculty of Biology, Medicine and Health, University of Manchester, Jean McFarlane Building, Manchester, M13 9PL. [2]Division of Psychology and Mental Health, School of Health Sciences, Faculty of Biology, Medicine and Health, University of Manchester, Jean McFarlane Building, Manchester, M13 9PL. [3]Department of Psychology, School of Human & Health Sciences, University of Huddersfield, Edith Key Building, Queensgate, Huddersfield, HD1 3DH. [4]5 Boroughs Partnership NHS Foundation Trust. [5]Lancashire Care NHS Foundation Trust, Preston, United Kingdom. [6]Centre for Mental Health and Risk, Division of Psychology and Mental Health, School of Health Sciences, Faculty of Biology, Medicine and Health, University of Manchester, Jean McFarlane Building, Manchester, M13 9PL. [7]Mental Health and Criminal Justice Group, University of Central Lancashire, Preston, UK. [8]Manchester Centre for Health Economics, Institute of Population Health, Division of Population Health, Health Services Research and Primary Care, University of Manchester, Jean McFarlane Building, Manchester, M13 9PL. [9]Offender Health Research Network, Division of

For peer review only - http://bmjopen.bmj.com/site/about/guidelines.xhtml

Psychology and Mental Health, School of Health Sciences, Faculty of Biology, Medicine and Health, University of Manchester, Jean McFarlane Building, Manchester, M13 9PL.

Figure 1. Consort diagram.

References

1 National Collaborating Centre for Mental Health. Self-Harm: The Short-Term Physical and Psychological Management and Secondary Prevention of Self-Harm in Primary and Secondary Care. London, England: National Institute for Clinical Excellence, 2004.

2 Hawton K, Arensman E, Townsend E, et al. Deliberate Self Harm: Systematic Review of Efficacy of Psychosocial and Pharmacological Treatments in Preventing Repetition. *Brit Med J* 1998;317(7156):441-7 doi:10.1136/bmj.317.7156.441 [published 15 August 1998].

3 Marzano L, Fazel S, Rivlin A, et al. Psychiatric disorders in women prisoners who have engaged in near-lethal self-harm: case–control study. *Br J Psychiatry* 2010;197:219-26 doi: 10.1192/bjp.bp.109.075424.

4 Ministry of Justice. Safer in custody statistics England and Wales: Deaths in custody to March 2016, Assaults and self-harm in custody to December 2015. London: Ministry of Justice, 2016.

5 Van Loey N, Van Son M. Psychopathology and psychological problems in patients with burn scars: epidemiology and management. *Am J Clin Dermatol* 2003;4:245-72 doi:10.2165/00128071-200304040-00004.

6 Brown B, McKenna S, Siddhi K, et al. The hidden cost of skin scars: quality of life after skin scarring. *J Plast Reconstr Aesthet Surg Open* 2008;61:1049–58 doi:10.1016/j.bjps.2008.03.020 [published 9 July 2008].

7 Rumsey N, Clarke A, Musa M. Altered Body Image: The Psychosocial Needs of Patients. *Br J Community Nurs* 2002;7(11):563-6 doi:10.12968/bjcn.2002.7.11.10886 [published November 2002].

8 Levy L, Emer J. Emotional Benefit of Cosmetic Camouflage in the Treatment of Facial Skin Conditions: Personal Experience and Review. *Clin Cosmet Investig Dermatol* 2012;5:173-82 doi: 10.2147/CCID.S33860 [published 1 November 2012].

For peer review only - http://bmjopen.bmj.com/site/about/guidelines.xhtml

9 Leibenluft E, Gardner D, Cowdry R. The inner experience of the borderline self-mutilator. *J Pers Disord* 1987;1:317-24 doi:10.1521/pedi.1987.1.4.317.

10 Chandler A. Narrating the self-injured body. *Med Humanit* 2014;40:111-16 doi:10.1136/medhum-2013-010488.

11 McMichael L. Skin Camouflage. *Brit Med J* 2012;344:d7921 doi:10.1136/bmj.d7921 [published 5 January 2012].

12 Holme S, Beattie P, Fleming C. Cosmetic Camouflage Advice Improves Quality of Life. *Br J Dermatol* 2002;147(5):946-9 doi:10.1046/j.1365-2133.2002.04900.x [published 1 November 2002].

13 Green L. The Effect of Skin Conditions on Patients' Quality of Life. *Nurs Stand* 2010;25(9):48-56 doi:10.7748/ns2010.11.25.9.48.c8078 [published 03 November 2010].

14 Kent G. Testing a Model of Disfigurement: Effects of a Skin Camouflage Service on Well-Being and Appearance Anxiety. *Psychol Health* 2002;17(3):377-86 doi:10.1080/08870440290029601 [published online 27 October 2010].

15 Tanioka M, Yamamoto Y, Kato M, et al. Camouflage for Patients with Vitiligo Vulgaris Improved their Quality of Life. *J Cosmet Dermatol* 2010;9(1):72-5 doi:10.1111/j.1473-2165.2010.00479.x [published 22 February 2010].

16 Hayashi N, Imori M, Yanagisawa M, et al. Make-up Improves the Quality of Life of Acne Patients without Aggravating Acne Eruptions during Treatments. *Eur J Dermatol* 2005;15(4):284-7.

17 McConochie L, Pearson E. Development of a Nurse-Led Skin Camouflage Clinic. *Nurs Stand* 2006;20(24):74, 6, 8 passim doi:10.7748/ns.20.24.74.s59 [published February 2006].

18 Walker T, Shaw J, Ranote S, et al. Medical Skin Camouflage: A Recovery Intervention for Female Prisoners who Self-Harm? *Crim Behav Ment Health* 2014;24(5):317-20 doi:10.1002/cbm.1943 [published 03 November 2014].

For peer review only - http://bmjopen.bmj.com/site/about/guidelines.xhtml

19 Kenning C, Cooper J, Short V, et al. Prison staff and women prisoner's views on self-harm; their implications for service delivery and development: A qualitative study. *Crim Behav Ment Health* 2010;20(4):274-84 doi:10.1002/cbm.777 [published online 05 July 2010].

20 Ranote S. Skin camouflage service: A first for mental health; Innovation to implementation. Talk presented at Self-harm in Women's Prisons Conference; 2016; University of Manchester.

21 Changing Faces. Skin Camouflage Practitioner Training Trainer's Practical Course Manual for COVER Project. London, England: Changing Faces, 2015.

22 Walker T, Shaw J, Turpin C, et al. The WORSHIP II study: a pilot of psychodynamic interpersonaltherapy with women offenders who self-harm. *J Forens Psychiatry Psychol* 2017;28(2):158-71 doi:10.1080/14789949.2017.1301529 [published online 08 March 2017].

23 Finlay AY, Khan GK. Dermatology Life Quality Index (DLQI)—a simple practical measure for routine clinical use. *Clin Exp Dermatol* 1994;19:210-6 doi:10.1111/j.1365-2230.1994.tb01167.x [published May 1994].

24 Rosenberg M. Society and the adolescent self-image. Princeton, N.J: Princeton University Press; 1965.

25 Gratz KL. Measurement of Deliberate Self-Harm: Preliminary Data on the Deliberate Self-Harm Inventory. *J Psychopathol Behav Assess* 2001;23(4):253-63 doi: 10.1023/A:1012779403943 [published December 2001].

26 Zanarini MC. Zanarini Rating Scale For Borderline Personality Disorder (ZAN-BPD): A Continuous Measure of DSM-IV Borderline Psychopathology. *J Pers Disord* 2003;17(3):233-242 doi: 10.1521/pedi.17.3.233.22147 [published July 2003].

27 Tennant R, Hiller L, Fishwick R, et al. The Warwick-Edinburgh Mental Well-being Scale (WEMWBS): development and UK validation. *Health Qual Life Outcomes* 2007;5(63) doi:10.1186/1477-7525-5-63 [published 27 November 2007].

28 Beck AT, Kovacs M, Weissman A. Assessment of Suicidal Intention - Scale for Suicide Ideation. *J Consult Clin Psych* 1979;47(2):343-52 doi:10.1037/0022-006X.47.2.343[published April 1979].

For peer review only - http://bmjopen.bmj.com/site/about/guidelines.xhtml

For peer review only

29 Beck AT, Steer RA, Brown GK. Manual for the BDI-II. San Antonio: TX: Psychological Corporation; 1996.

30 Wang YP, Gorenstein C. Psychometric properties of the Beck Depression Inventory-II: a comprehensive review. *Rev Bras Psiquiatr* 2013;35:416-31 doi:10.1590/1516-4446-2012-1048 [published online 23 December 2013].

31 Beck AT, Weissman A, Lester D, et al. The Measurement of Pessimism: The Hopelessness Scale. *J Consult Clin Psych* 1974;42(6):861-5 doi:10.1037/h0037562 [published December 1974].

32 Boduszek D, Shevlin M, Mallett J, et al. Dimensionality and construct validity of the Rosenberg Self-Esteem Scale within a sample of recidivistic prisoners. *J Crim Psychol* 2012;2(1):19-25 doi: 10.1108/20093821211210468.

33 Gusi N, Olivares PR, Rajendram R. The EQ-5D Health-Related Quality of Life Questionnaire. Springer. USA, New York. 2010:87-99.

34 Haywood KL, Garratt AM, Dziedzic K, et al. Generic measures of health-related quality of life in ankylosing spondylitis: reliability, validity and responsiveness. *Rheumatology* 2002;41:1380-87 doi: 10.1093/rheumatology/41.12.1380 [published 01 December 2002].

35 Ware JJ, Kosinski M, Keller SD. A 12-Item Short-Form Health Survey: construction of scales and preliminary tests of reliability and validity. *Med Care* 1996;34(3):220-33 doi:10.2307/3766749 [published April 1996].

36 Barrett B, Byford S. Collecting service use data for economic evaluation in DSPD populations: development of the Secure Facilities Service Use Schedule. *Br J Psychiatry* 2007;190(suppl. 49):s79-s79 doi:10.1192/bjp.190.5.s75 [published online 03 January 2018].

37 Braun V, Clark V. Using Thematic Analysis in Psychology. *Qual Res Psychol* 2006;3(2):77-101 doi:10.1191/1478088706qp063oa [published online 21 July 2008].

38 Boyatzis R. Transforming Qualitative Information: Thematic Analysis and Code Development. California: Sage 1998.

For peer review only - http://bmjopen.bmj.com/site/about/guidelines.xhtml

3[illegible] M[illegible] N, P[illegible] [illegible] Qual[illegible] research: R[illegible]go[illegible] and qua[illegible] research. [illegible] 1[illegible]5;[illegible]11:1[illegible]9-[illegible]2
doi:[illegible]

For peer review only

For peer review only - http://bmjopen.bmj.com/site/about/guidelines.xhtml

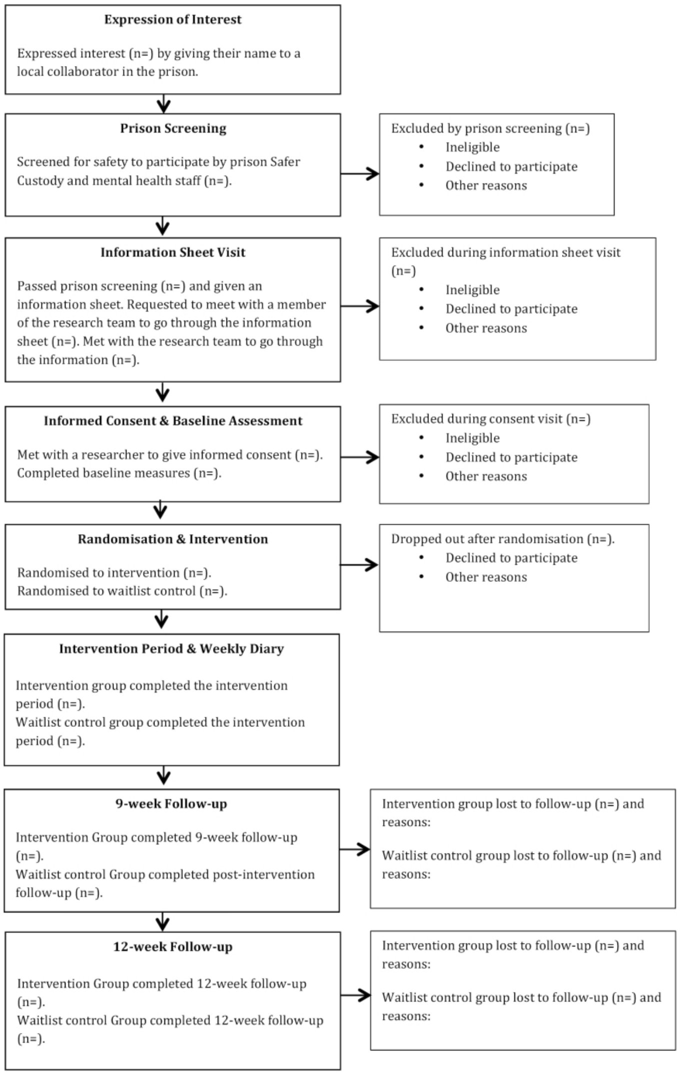

Figure 1. Consort diagram.

239x310mm (600 x 600 DPI)

SPIRIT — STANDARD PROTOCOL ITEMS: RECOMMENDATIONS FOR INTERVENTIONAL TRIALS

SPIRIT 2013 Checklist: Recommended items to address in a clinical trial protocol and related documents*

Section/item	Item No	Description
**Administrative information**		
Title	1	Descriptive title identifying the study design, population, interventions, and, if applicable, trial acronym
Trial registration	2a	Trial identifier and registry name. If not yet registered, name of intended registry
	2b	All items from the World Health Organization Trial Registration Data Set
Protocol version	3	Date and version identifier
Funding	4	Sources and types of financial, material, and other support
Roles and responsibilities	5a	Names, affiliations, and roles of protocol contributors
	5b	Name and contact information for the trial sponsor
	5c	Role of study sponsor and funders, if any, in study design; collection, management, analysis, and interpretation of data; writing of the report; and the decision to submit the report for publication, including whether they will have ultimate authority over any of these activities
	5d	Composition, roles, and responsibilities of the coordinating centre, steering committee, endpoint adjudication committee, data management team, and other individuals or groups overseeing the trial, if applicable (see Item 21a for data monitoring committee)
**Introduction**		
Background and rationale	6a	Description of research question and justification for undertaking the trial, including summary of relevant studies (published and unpublished) examining benefits and harms for each intervention
	6b	Explanation for choice of comparators
Objectives	7	Specific objectives or hypotheses
Trial design	8	Description of trial design including type of trial (eg, parallel group, crossover, factorial, single group), allocation ratio, and framework (eg, superiority, equivalence, noninferiority, exploratory)

For peer review only

Methods: Participants, interventions, and outcomes

Study setting	9	Description of study settings (eg, community clinic, academic hospital) and list of countries where data will be collected. Reference to where list of study sites can be obtained
Eligibility criteria	10	Inclusion and exclusion criteria for participants. If applicable, eligibility criteria for study centres and individuals who will perform the interventions (eg, surgeons, psychotherapists)
Interventions	11a	Interventions for each group with sufficient detail to allow replication, including how and when they will be administered
	11b	Criteria for discontinuing or modifying allocated interventions for a given trial participant (eg, drug dose change in response to harms, participant request, or improving/worsening disease)
	11c	Strategies to improve adherence to intervention protocols, and any procedures for monitoring adherence (eg, drug tablet return, laboratory tests)
	11d	Relevant concomitant care and interventions that are permitted or prohibited during the trial
Outcomes	12	Primary, secondary, and other outcomes, including the specific measurement variable (eg, systolic blood pressure), analysis metric (eg, change from baseline, final value, time to event), method of aggregation (eg, median, proportion), and time point for each outcome. Explanation of the clinical relevance of chosen efficacy and harm outcomes is strongly recommended
Participant timeline	13	Time schedule of enrolment, interventions (including any run-ins and washouts), assessments, and visits for participants. A schematic diagram is highly recommended (see Figure)
Sample size	14	Estimated number of participants needed to achieve study objectives and how it was determined, including clinical and statistical assumptions supporting any sample size calculations
Recruitment	15	Strategies for achieving adequate participant enrolment to reach target sample size

Methods: Assignment of interventions (for controlled trials)

Allocation:

Sequence generation	16a	Method of generating the allocation sequence (eg, computer-generated random numbers), and list of any factors for stratification. To reduce predictability of a random sequence, details of any planned restriction (eg, blocking) should be provided in a separate document that is unavailable to those who enrol participants or assign interventions

Allocation concealment mechanism	16b	Mechanism of implementing the allocation sequence (eg, central telephone; sequentially numbered, opaque, sealed envelopes), describing any steps to conceal the sequence until interventions are assigned
Implementation	16c	Who will generate the allocation sequence, who will enrol participants, and who will assign participants to interventions
Blinding (masking)	17a	Who will be blinded after assignment to interventions (eg, trial participants, care providers, outcome assessors, data analysts), and how
	17b	If blinded, circumstances under which unblinding is permissible, and procedure for revealing a participant's allocated intervention during the trial
**Methods: Data collection, management, and analysis**		
Data collection methods	18a	Plans for assessment and collection of outcome, baseline, and other trial data, including any related processes to promote data quality (eg, duplicate measurements, training of assessors) and a description of study instruments (eg, questionnaires, laboratory tests) along with their reliability and validity, if known. Reference to where data collection forms can be found, if not in the protocol
	18b	Plans to promote participant retention and complete follow-up, including list of any outcome data to be collected for participants who discontinue or deviate from intervention protocols
Data management	19	Plans for data entry, coding, security, and storage, including any related processes to promote data quality (eg, double data entry; range checks for data values). Reference to where details of data management procedures can be found, if not in the protocol
Statistical methods	20a	Statistical methods for analysing primary and secondary outcomes. Reference to where other details of the statistical analysis plan can be found, if not in the protocol
	20b	Methods for any additional analyses (eg, subgroup and adjusted analyses)
	20c	Definition of analysis population relating to protocol non-adherence (eg, as randomised analysis), and any statistical methods to handle missing data (eg, multiple imputation)
**Methods: Monitoring**		
Data monitoring	21a	Composition of data monitoring committee (DMC); summary of its role and reporting structure; statement of whether it is independent from the sponsor and competing interests; and reference to where further details about its charter can be found, if not in the protocol. Alternatively, an explanation of why a DMC is not needed

	21b	Description of any interim analyses and stopping guidelines, including who will have access to these interim results and make the final decision to terminate the trial
Harms	22	Plans for collecting, assessing, reporting, and managing solicited and spontaneously reported adverse events and other unintended effects of trial interventions or trial conduct
Auditing	23	Frequency and procedures for auditing trial conduct, if any, and whether the process will be independent from investigators and the sponsor
**Ethics and dissemination**		
Research ethics approval	24	Plans for seeking research ethics committee/institutional review board (REC/IRB) approval
Protocol amendments	25	Plans for communicating important protocol modifications (eg, changes to eligibility criteria, outcomes, analyses) to relevant parties (eg, investigators, REC/IRBs, trial participants, trial registries, journals, regulators)
Consent or assent	26a	Who will obtain informed consent or assent from potential trial participants or authorised surrogates, and how (see Item 32)
	26b	Additional consent provisions for collection and use of participant data and biological specimens in ancillary studies, if applicable
Confidentiality	27	How personal information about potential and enrolled participants will be collected, shared, and maintained in order to protect confidentiality before, during, and after the trial
Declaration of interests	28	Financial and other competing interests for principal investigators for the overall trial and each study site
Access to data	29	Statement of who will have access to the final trial dataset, and disclosure of contractual agreements that limit such access for investigators
Ancillary and post-trial care	30	Provisions, if any, for ancillary and post-trial care, and for compensation to those who suffer harm from trial participation
Dissemination policy	31a	Plans for investigators and sponsor to communicate trial results to participants, healthcare professionals, the public, and other relevant groups (eg, via publication, reporting in results databases, or other data sharing arrangements), including any publication restrictions
	31b	Authorship eligibility guidelines and any intended use of professional writers
	31c	Plans, if any, for granting public access to the full protocol, participant-level dataset, and statistical code

Appendices

Informed consent materials	32	Model consent form and other related documentation given to participants and authorised surrogates
Biological specimens	33	Plans for collection, laboratory evaluation, and storage of biological specimens for genetic or molecular analysis in the current trial and for future use in ancillary studies, if applicable

*It is strongly recommended that this checklist be read in conjunction with the SPIRIT 2013 Explanation & Elaboration for important clarification on the items. Amendments to the protocol should be tracked and dated. The SPIRIT checklist is copyrighted by the SPIRIT Group under the Creative Commons "Attribution-NonCommercial-NoDerivs 3.0 Unported" license.

For peer review only
